# Synergistic Antitumor Effects of Etoposide and Curcumin in Ovarian Cancer Cells

**DOI:** 10.3390/biomedicines14030509

**Published:** 2026-02-25

**Authors:** Yunus Çavuş, Senem Alkan Akalın, Yasemin Afşin, Veysel Toprak, İlhan Özdemir, Mehmet Cudi Tuncer, Şamil Öztürk

**Affiliations:** 1 Department of Gynecology and Obstetrics, Private Bower Hospital, Diyarbakır 21100, Turkey; dryunuscavus34@gmail.com; 2 Department of Gynecology and Obstetrics, Private Medical Practise, Bursa 16990, Turkey; drsakalin@hotmail.com; 3 Department of Gynecology and Obstetrics, Private Batman Life Hospital, Batman 72040, Turkey; dryaseminafsin@outlook.com; 4 Department of Gynecology and Obstetrics, Faculty of Medicine, Private Metrolife Hospital, Şanlıurfa 63320, Turkey; drveysel21@outlook.com; 5 Department of Histology Embryology, Faculty of Medicine, Kahramanmaraş Sütçü İmam University, Kahramanmaraş 46100, Turkey; ilhanozdemir25@yandex.com; 6 Department of Anatomy, Faculty of Medicine, Dicle University, Diyarbakır 21280, Turkey; drcudi@hotmail.com; 7 Vocational School of Health Services, Çanakkale Onsekiz Mart University, Çanakkale 17100, Turkey

**Keywords:** curcumin, etoposide, ovarian cancer, cell death, apoptosis, cancer

## Abstract

**Background**: Overcoming therapeutic resistance and tumor microenvironment-mediated survival remains a major challenge in ovarian cancer treatment. This study investigated the individual and combined antitumor effects of etoposide and curcumin in human ovarian cancer models, with emphasis on synergistic interactions, apoptosis induction, tumor microenvironment modulation, and oxidative stress-associated mechanisms. We hypothesized that the combination of etoposide and curcumin exerts enhanced antitumor activity through an integrated mechanism involving DNA damage-associated apoptotic signaling, tumor microenvironment modulation, and ROS-associated cellular stress, rather than through a single dominant pathway. **Methods**: Cell viability was assessed using the MTT assay, and drug–drug interactions were quantitatively evaluated using the Chou–Talalay and Bliss independence models. Apoptosis was analyzed by Annexin V/propidium iodide flow cytometry, caspase-8/9 activity assays, and cell cycle analysis. Transcriptional regulation of apoptosis- and microenvironment-related genes was examined by quantitative real-time PCR. Inflammatory and angiogenic cytokines were measured by ELISA. Therapeutic efficacy was further validated in three-dimensional (3D) tumor spheroid models using morphological assessment and adenosine triphosphate (ATP)-based viability assays. The contribution of reactive oxygen species (ROS) was evaluated using antioxidant pretreatment. **Results**: The etoposide–curcumin combination demonstrated significantly enhanced antiproliferative and pro-apoptotic effects compared with either agent alone, with consistent synergism observed across quantitative interaction models. Combination treatment increased apoptotic cell death, activated both intrinsic and extrinsic apoptotic pathways, and induced G2/M cell cycle arrest. In parallel, inflammatory and angiogenic signaling was markedly suppressed at both transcriptional and protein levels. These effects were preserved and amplified in 3D tumor spheroid models, where combination therapy induced pronounced spheroid shrinkage and reduced viability. Antioxidant pretreatment partially attenuated ROS generation and cytotoxicity, indicating that oxidative stress contributes to, but does not fully account for, the observed antitumor effects. **Conclusions**: The combination of etoposide and curcumin exerts synergistic and multi-layered antitumor effects in ovarian cancer models by integrating apoptosis induction, tumor microenvironment modulation, and ROS-associated mechanisms. These findings support further preclinical evaluation of this combination as a rational therapeutic strategy for ovarian cancer.

## 1. Introduction

Ovarian cancer remains the most lethal gynecological malignancy, largely due to the fact that the majority of patients are diagnosed at advanced stages of the disease [1]. Despite advances in surgical techniques and the widespread use of platinum- and taxane-based chemotherapy, disease recurrence and the development of chemoresistance continue to represent major clinical challenges [2]. These limitations are driven by multiple interconnected mechanisms, including enhanced DNA damage repair capacity, dysregulation of apoptotic signaling pathways, and pro-survival cues derived from the tumor microenvironment [3]. Consequently, there is an urgent need for novel therapeutic strategies that can enhance the efficacy of existing chemotherapeutic agents while minimizing toxicity and resistance.

Etoposide is a well-established chemotherapeutic agent that exerts its cytotoxic effects primarily through inhibition of topoisomerase II, leading to DNA strand breaks and apoptosis induction [4]. However, its clinical utility is frequently constrained by dose-limiting toxicities and the emergence of resistance during prolonged treatment. Combining conventional chemotherapeutics with bioactive natural compounds has emerged as a promising approach to overcome these limitations and improve therapeutic outcomes [5,6,7]. In this context, curcumin, the principal polyphenolic compound derived from Curcuma longa, has attracted considerable attention due to its broad-spectrum anticancer properties [8]. Curcumin has been shown to inhibit cancer cell proliferation, promote apoptotic cell death [9,10], modulate inflammatory signaling, and suppress angiogenesis [11]. Importantly, accumulating evidence suggests that curcumin can act synergistically with standard chemotherapeutic agents, enhancing anticancer efficacy while potentially reducing systemic toxicity [9].

Cell culture-based experimental models are indispensable for investigating the efficacy and mechanisms of anticancer drug combinations. However, contemporary research standards extend beyond simple viability-based cytotoxicity assays. A rigorous experimental framework requires quantitative assessment of drug–drug interactions [12], validation of apoptotic progression through coordinated biological endpoints such as cell cycle regulation and caspase activation, and molecular characterization of key signaling pathways governing cell fate decisions. In parallel, increasing attention has been directed toward the tumor microenvironment, as inflammatory cytokines such as interleukin-6 (IL-6) and tumor necrosis factor-α (TNF-α), along with angiogenic mediators such as vascular endothelial growth factor (VEGF), play critical roles in tumor progression, therapy resistance, and metastatic potential [13,14].

Accordingly, the objective of the present study was to comprehensively investigate the combined effects of etoposide and curcumin in OVCAR3 and SKOV3 human ovarian cancer cell lines using an integrated and mechanistically oriented approach. The synergistic interaction between the two agents was quantitatively evaluated, apoptosis induction was validated at both cellular and transcriptional levels, and the ability of the combination to modulate tumor microenvironment-related signaling was examined [11,15].

Based on these considerations, we hypothesized that the combination of etoposide and curcumin exerts enhanced antitumor activity through an integrated and multifactorial biological framework involving DNA damage-associated apoptotic signaling, modulation of tumor microenvironment-related pathways, and ROS-associated cellular stress, rather than through a single dominant pathway. Furthermore, to evaluate the biological relevance of this integrated response beyond conventional two-dimensional cultures, we employed 3D tumor spheroid models together with ROS-modulation experiments. This experimental framework was designed to approximate key aspects of in vivo tumor biology and to assess the contribution of oxidative stress within a broader apoptotic and microenvironmental context. Collectively, this study aims to provide a robust preclinical and hypothesis-generating framework supporting the rational development of combination strategies for ovarian cancer treatment.

## 2. Materials and Methods

### 2.1. Chemicals and Reagents

Etoposide and curcumin were obtained from Sigma-Aldrich (St. Louis, MO, USA). All other reagents and chemicals used throughout the study were of analytical grade or higher purity and were purchased from commercially available suppliers. Stock solutions of etoposide and curcumin were freshly prepared in dimethyl sulfoxide (DMSO) at appropriate concentrations and stored at −20 °C in light-protected conditions to preserve chemical stability.

Prior to each experiment, working solutions were freshly prepared by diluting the stock solutions in complete cell culture medium. To exclude solvent-related cytotoxic effects and ensure experimental consistency, the final concentration of DMSO in all treatment and control conditions was maintained below 0.1% (*v*/*v*). Vehicle control groups containing equivalent DMSO concentrations were included in all experiments and processed in parallel with drug-treated samples. For combination treatments, the cumulative DMSO concentration resulting from the simultaneous administration of etoposide and curcumin was carefully calculated and maintained below 0.1% (*v*/*v*), matching the vehicle concentrations used in single-agent treatments.

### 2.2. Cell Culture and Experimental Conditions

Human ovarian adenocarcinoma cell lines OVCAR3 and SKOV3 were obtained from the American Type Culture Collection (ATCC, Manassas, VA, USA). OVCAR3 cells were maintained in RPMI-1640 medium, whereas SKOV3 cells were cultured in McCoy’s 5A medium. Both media were supplemented with 10% (*v*/*v*) fetal bovine serum (FBS; Gibco, Thermo Fisher Scientific, Waltham, MA, USA) and 1% (*v*/*v*) penicillin–streptomycin solution (Gibco) to prevent bacterial contamination.

OVCAR3 and SKOV3 cell lines were deliberately selected to model biologically and molecularly distinct ovarian cancer contexts rather than a single disease entity. Extensive genomic and immunophenotypic studies have demonstrated that epithelial ovarian cancer comprises multiple histotype-specific diseases with distinct molecular drivers and therapeutic vulnerabilities, underscoring the need for stratified in vitro model selection [16,17]. Within this framework, OVCAR3 aligns with high-grade serous ovarian carcinoma, characterized by p53 pathway disruption and stress-responsive apoptotic signaling typical of this histotype [16,18]. In contrast, SKOV3 segregates from bona fide high-grade serous models at both molecular and phenotypic levels and exhibits distinct proteomic, phospho-signaling, and adaptive stress-response profiles [16,19]. Large-scale comparative analyses further indicate that such differences translate into heterogeneity in drug sensitivity, apoptotic competence, and tumor microenvironment-associated signaling across ovarian cancer models [17,19]. Accordingly, the combined use of OVCAR3 and SKOV3 provides a rational framework to evaluate the antitumor effects of the etoposide–curcumin combination across heterogeneous ovarian cancer contexts with distinct molecular ancestry and adaptive capacity.

Cells were cultured in a humidified incubator at 37 °C under an atmosphere containing 5% CO_2_ and were routinely monitored for morphology and growth characteristics. Culture medium was replaced every 2–3 days, and cells were passaged using standard trypsinization procedures upon reaching appropriate confluence. For all experiments, cells in the logarithmic growth phase with approximately 70–80% confluence were used to ensure uniform metabolic activity and reproducibility across experimental conditions.

### 2.3. Cell Viability Assay and Quantitative Combination Index (CI) Analysis

Cell viability was evaluated using the standard MTT [3-(4,5-dimethylthiazol-2-yl)-2,5-diphenyltetrazolium bromide] colorimetric assay following 24, 48, and 72 h of drug exposure to capture both early and time-dependent cytotoxic effects. Briefly, cells were seeded into 96-well plates at optimized densities and treated with increasing concentrations of etoposide or curcumin, either alone or in combination. After the indicated incubation periods, MTT reagent was added to each well, and the resulting formazan crystals were solubilized prior to spectrophotometric measurement. Cell viability was expressed as a percentage relative to vehicle-treated control cells.

Dose–response curves were generated for each compound individually, and half-maximal inhibitory concentration (IC_50_) values were calculated using nonlinear regression analysis. For combination experiments, etoposide and curcumin were applied in fixed-ratio serial dilutions based on their respective IC_50_ values to enable accurate assessment of drug–drug interactions across a range of fractional effects.

Importantly, IC_50_ values were used as operational reference points to define fixed-ratio combination conditions rather than as absolute pharmacological endpoints. Accordingly, subsequent interaction analyses were based on dose–effect relationships evaluated across a broad range of fractional effects and concentration levels, thereby reducing reliance on a single concentration estimate and minimizing potential uncertainty arising from incomplete upper or lower response plateaus in individual dose–response curves.

CI analysis was performed over a fractional effect (Fa) range of 0.3–0.8, which corresponds to biologically relevant and experimentally reliable response levels commonly recommended for Chou–Talalay-based synergy assessment. Extreme low (Fa < 0.3) and high (Fa > 0.8) effect levels were not emphasized due to increased variability and reduced robustness of CI estimation at response extremes, particularly in systems exhibiting non-ideal dose–response curvature.

CI values were calculated for each individual fractional effect within this range, and mean CI values are reported solely as a descriptive summary to facilitate comparison between cell lines, rather than as a substitute for effect-level–specific interaction analysis. Effect-dependent CI distributions are therefore explicitly presented in Section 3 to avoid obscuring potential heterogeneity in interaction behavior across different response levels.

The nature of the interaction between etoposide and curcumin was quantitatively evaluated using the Chou–Talalay method, which allows distinction between synergistic, additive, and antagonistic effects based on the median-effect principle. CI values were calculated using CompuSyn software (version 1.0, ComboSyn, Inc., Paramus, NJ, USA). CI values < 1 indicated synergism, CI = 1 indicated an additive effect, and CI values > 1 indicated antagonism [12]. This quantitative approach provided a robust and reproducible framework for assessing the interaction profile of the combination treatment.

### 2.4. Bliss Independence Model for Dose–Response Interaction Analysis

To independently validate the interaction profile obtained using the Chou–Talalay approach and to characterize dose-dependent interaction patterns in greater detail, combination effects were further analyzed using the Bliss independence model. This model is based on the assumption that two agents act independently through distinct mechanisms and therefore defines an expected additive effect derived from the individual activities of each compound.

According to the Bliss independence principle, the expected additive effect of the combination (E_exp) was calculated from the fractional inhibitory effects of the single agents (f_A and f_B) using the following equation:

$$E_{\exp}=f_{A}+f_{B}-(f_{A}\times f_{B})$$

The experimentally observed effect of the drug combination (E_obs) was then compared with the expected additive effect, and the deviation from additivity was expressed as the Bliss synergy score (ΔE), calculated as:

$$\Delta E=E_{\mathrm{obs}}-E_{\exp}$$

Positive ΔE values were interpreted as synergistic interactions, values close to zero indicated additive effects, and negative ΔE values reflected antagonistic interactions [20].

Importantly, Bliss independence analysis was performed across a defined concentration matrix spanning sub-IC_50_, IC_50_-equivalent, and supra-IC_50_ dose levels of both agents, enabling assessment of interaction behavior across multiple effect intensities rather than at a single concentration point. This approach allowed identification of dose-dependent differences in interaction profiles between cell lines. Bliss synergy scores were therefore interpreted in a context-dependent manner, with particular emphasis on the consistency and robustness of positive ΔE values across the tested dose matrix. In this framework, OVCAR3 cells exhibited predominantly positive Bliss scores across a broad concentration range, indicating dose-consistent synergistic interactions, whereas SKOV3 cells demonstrated a more moderate and concentration-restricted synergistic response, with attenuation or loss of synergy at higher curcumin concentrations. Accordingly, Bliss independence analysis was used not to assert uniform synergy across all dose levels, but rather to delineate cell line–specific and dose-dependent interaction patterns and to complement CI-based analyses with an independent mathematical model.

Bliss synergy scores were calculated for all tested dose combinations and visualized using heatmap representations to enable systematic comparison of interaction patterns across the full concentration matrix. This complementary analytical approach provided an independent and mathematically grounded validation of synergistic, additive, or antagonistic effects observed for the etoposide–curcumin combination.

### 2.5. Flow Cytometric Analysis of Apoptosis and Cell Cycle Distribution

Following 48 h of treatment, cells were harvested, washed with cold phosphate-buffered saline (PBS), and subjected to apoptosis analysis using Annexin V–fluorescein isothiocyanate (FITC) and PI staining (BD Biosciences, San Jose, CA, USA), in accordance with the manufacturer’s instructions. Stained cells were analyzed by flow cytometry using a BD FACSVerse system (BD Biosciences, San Jose, CA, USA). Cell populations were classified as viable (Annexin V^−^/PI^−^), early apoptotic (Annexin V^+^/PI^−^), late apoptotic/necrotic (Annexin V^+^/PI^+^), or necrotic (Annexin V^−^/PI^+^). The extent of apoptosis was calculated as the combined proportion of early and late apoptotic cells.

For cell cycle analysis, cells were collected after treatment, fixed in cold 70% ethanol, and stored at −20 °C to ensure proper DNA preservation. Prior to analysis, fixed cells were washed, treated with RNase A to remove residual RNA, and stained with PI. DNA content was subsequently analyzed by flow cytometry, and cell cycle distribution was determined based on fluorescence intensity, allowing quantification of cell populations in the G0/G1, S, and G2/M phases.

### 2.6. Measurement of Caspase-8 and Caspase-9 Activities

Activation of apoptosis-initiating caspases was assessed to distinguish between extrinsic and intrinsic apoptotic pathway involvement following treatment. Caspase-8 activity, representing activation of the death receptor-mediated extrinsic pathway, and caspase-9 activity, reflecting mitochondrial intrinsic pathway activation, were quantified using luminescence-based assay kits (Caspase-Glo^®^ 8 and Caspase-Glo^®^ 9 Assay Systems; Promega, Madison, WI, USA).

OVCAR3 and SKOV3 cells were treated with etoposide, curcumin, or their combination for 48 h. Following treatment, the appropriate Caspase-Glo^®^ reagent was added directly to each well according to the manufacturer’s instructions, allowing cell lysis and generation of a luminescent signal proportional to caspase activity. After incubation at room temperature in the dark, luminescence was measured using a microplate reader.

Caspase activity values were normalized to those of vehicle-treated control cells and expressed as fold change relative to control, enabling quantitative comparison of caspase activation across treatment conditions.

### 2.7. Quantification of Inflammatory and Angiogenic Cytokines by ELISA

To assess the effects of etoposide and curcumin treatments on tumor microenvironment-associated signaling, culture supernatants from OVCAR3 and SKOV3 cells were collected after 48 h of treatment. Following removal of cellular debris by brief centrifugation, supernatants were stored at −80 °C until analysis to preserve cytokine stability.

The concentrations of human interleukin-6 (IL-6), TNF-α, and VEGF were quantified using commercially available enzyme-linked immunosorbent assay (ELISA) kits (R&D Systems, Minneapolis, MN, USA) according to the manufacturer’s protocols. Optical density values were measured using a microplate reader at the specified wavelengths, and cytokine concentrations were calculated from standard curves generated using recombinant cytokine standards.

Cytokine levels were expressed as absolute concentrations and subsequently normalized to control groups to allow quantitative comparison of treatment-induced changes in inflammatory and angiogenic signaling.

### 2.8. Quantitative Real-Time Polymerase Chain Reaction (qRT-PCR) Analysis

Total RNA was isolated from treated and control OVCAR3 and SKOV3 cells using TRIzol reagent (Invitrogen, Thermo Fisher Scientific, Waltham, MA, USA) according to the manufacturer’s instructions. RNA concentration and purity were assessed spectrophotometrically, and samples with acceptable purity ratios were used for subsequent analyses. Complementary DNA (cDNA) was synthesized from equal amounts of total RNA using a High-Capacity cDNA Reverse Transcription Kit (Applied Biosystems, Foster City, CA, USA).

Quantitative real-time PCR (qRT-PCR) analysis was performed using SYBR Green Master Mix (Roche Diagnostics, Mannheim, Germany) on a LightCycler^®^ 480 II Real-Time PCR System (Roche). The mRNA expression levels of the target genes BAX, BCL2, TP53, and CASP3 were quantified. Glyceraldehyde-3-phosphate dehydrogenase (GAPDH) and β-actin were used as internal reference genes. The expression stability of these reference genes was verified across experimental conditions, and normalization was performed using the geometric mean of GAPDH and β-actin expression values to improve normalization accuracy and reduce potential bias associated with the use of a single reference gene.

Relative gene expression levels were calculated using the comparative threshold cycle (2^−ΔΔCT^) method, with results expressed as fold change relative to control samples. This approach enabled reliable comparison of transcriptional alterations induced by etoposide, curcumin, and their combination across experimental conditions.

### 2.9. In Silico Bioinformatics Analysis and Target Network Exploration

Comprehensive in silico and bioinformatics analyses were performed to support the experimental findings and to provide mechanistic insight into the potential synergistic effects of etoposide and curcumin. These analyses aimed to (i) predict the pharmacological and toxicological properties of the compounds, (ii) identify putative molecular targets and shared signaling nodes, (iii) explore protein–protein interaction networks underlying combination effects, and (iv) define the biological pathways and functional processes associated with the identified targets.

By integrating target prediction, network-based analysis, and functional enrichment approaches, the bioinformatics framework was used to generate hypothesis-driven mechanistic interpretations of the observed apoptotic, cell cycle–regulatory, and tumor microenvironment–modulating effects. Importantly, these in silico analyses were employed as supportive and hypothesis-generating tools to contextualize the experimental findings, rather than to provide direct experimental validation of specific molecular targets. This integrative strategy enabled contextualization of the experimental results within broader signaling networks relevant to ovarian cancer biology and combination therapy response.

#### 2.9.1. In Silico Prediction of Pharmacokinetic and Drug-likeness Properties

The pharmacokinetic and drug-likeness properties of etoposide and curcumin were evaluated using the SwissADME web-based platform (http://www.swissadme.ch; accessed on 10 January 2026). This analysis was performed to obtain predictive information regarding absorption, distribution, and overall suitability of the compounds for biological applications.

Key parameters assessed included compliance with Lipinski’s rule of five, predicted oral bioavailability, gastrointestinal absorption, and P-glycoprotein substrate status. These parameters were selected to provide an integrated overview of the physicochemical and pharmacokinetic characteristics that may influence cellular uptake, intracellular availability, and potential drug–drug interactions.

The SwissADME analysis (http://www.swissadme.ch; accessed on 10 January 2026) was used as a supportive, hypothesis-generating tool to contextualize experimental findings and did not replace experimental pharmacokinetic evaluation.

#### 2.9.2. Protein–Protein Interaction (PPI) Network Construction and Analysis

Potential protein targets of etoposide and curcumin were predicted based on two-dimensional structural similarity using the SwissTargetPrediction server (http://www.swisstargetprediction.ch; accessed on 10 January 2026). High-probability targets identified for each compound were combined to generate a common target set representing potential molecular points of convergence for combination therapy.

PPI analysis of the common target set was performed using the STRING database (version 12.0; https://string-db.org; accessed on 10 January 2026). Only interactions with a high confidence score (>0.700) were included to ensure robustness and biological relevance of the network. The resulting interaction network was subsequently imported into Cytoscape software (version 3.10.0) for visualization and topological analysis.

Network topology parameters, including degree centrality and betweenness centrality, were calculated to identify hub genes and key regulatory nodes within the network. These hub proteins were considered potential mediators of the observed synergistic effects and provided a systems-level framework for interpreting the experimental findings.

#### 2.9.3. Functional and Pathway Enrichment Analysis (GO and KEGG)

To explore the biological relevance of the predicted common target proteins and to contextualize them within known cellular processes and signaling pathways, functional enrichment analyses were performed using Gene Ontology (GO) and Kyoto Encyclopedia of Genes and Genomes (KEGG) databases.

GO enrichment analysis was conducted to identify significantly overrepresented terms across the Biological Process (BP), Cellular Component (CC), and Molecular Function (MF) categories associated with the common target gene set. In parallel, KEGG pathway enrichment analysis was performed to determine major signaling pathways in which these targets are involved, including pathways related to apoptosis, cell survival, and stress response, such as the p53 signaling pathway, PI3K–Akt signaling pathway, apoptosis, and HIF-1 signaling pathway.

All enrichment analyses were performed using the clusterProfiler R package (version 4.6.0). Statistical significance was determined using false discovery rate (FDR)–adjusted *p*-values, with an FDR threshold of <0.05 considered statistically significant.

### 2.10. 3D Tumor Spheroid Culture and Treatment

#### 2.10.1. Formation of 3D Tumor Spheroids

To evaluate treatment effects under conditions that more closely mimic the in vivo tumor microenvironment, 3D tumor spheroid models were established. Ovarian cancer cells were harvested during the logarithmic growth phase and resuspended in complete culture medium. Cells were seeded into ultra-low attachment (ULA) 96-well round-bottom plates (Corning Inc., Corning, NY, USA) at a density of 3 × 10^3^ cells per well in a final volume of 200 µL.

Cells were incubated at 37 °C in a humidified atmosphere containing 5% CO_2_, allowing spontaneous spheroid formation. Compact and uniformly shaped spheroids formed within 48–72 h, as confirmed by inverted light microscopy. Only spheroids exhibiting regular spherical morphology were included in subsequent experiments to ensure experimental consistency.

#### 2.10.2. Drug Treatment of 3D Tumor Spheroids

Following spheroid formation, culture medium was carefully replaced with fresh medium containing curcumin, etoposide, or their combination (etoposide + curcumin). Drug concentrations were selected based on IC_50_ values obtained from 2D monolayer experiments and were modestly increased (approximately 20–30%) to account for reduced effective drug exposure in 3D spheroid systems, which is attributed to diffusion gradients, limited drug penetration, and enhanced cell–cell interactions. Such empirical dose adjustments are commonly applied in 3D tumor spheroid models to achieve biologically comparable treatment effects to those observed in 2D cultures [21,22].

The following experimental groups were established:

- Control (vehicle-treated);
- Curcumin-treated;
- Etoposide-treated;
- Etoposide + curcumin-treated.

Spheroids were incubated with the indicated treatments for 72 h prior to downstream analyses. Vehicle concentrations were kept constant across all experimental groups to exclude solvent-related effects.

#### 2.10.3. Bright-Field Imaging and Spheroid Size Quantification

Bright-field images of 3D tumor spheroids were acquired using an inverted microscope equipped with a digital camera (Olympus Corporation, Tokyo, Japan) at 10× magnification. Images were captured using identical optical and acquisition settings for all experimental groups to allow direct comparison.

Spheroid size was quantified by measuring spheroid diameter using ImageJ software (version 1.53; National Institutes of Health, Bethesda, MD, USA) Spheroid diameter was defined as the mean of two perpendicular measurements obtained at the widest points of the spheroid. For each experimental condition, at least three independent biological experiments were performed, with a minimum of three spheroids analyzed per condition in each experiment, and mean spheroid diameter values were calculated.

#### 2.10.4. Cell Viability Assessment in 3D Tumor Spheroids

Cell viability within 3D tumor spheroids was assessed using the CellTiter-Glo^®^ 3D Cell Viability Assay (Promega, Madison, WI, USA), specifically optimized for 3D culture systems. The assay was performed according to the manufacturer’s instructions.

Briefly, an equal volume of CellTiter-Glo^®^ 3D reagent was added directly to each well containing spheroids, followed by orbital shaking to ensure efficient spheroid lysis and ATP release. Luminescence was measured using a microplate reader, and viability values were expressed as a percentage relative to vehicle-treated control spheroids.

#### 2.10.5. Live/Dead Fluorescence Staining of 3D Tumor Spheroids

To visualize treatment-induced cytotoxicity within 3D tumor spheroids, live/dead fluorescence staining was performed using Calcein-AM and Ethidium homodimer-1 (EthD-1). Following treatment, spheroids were incubated with Calcein-AM (2 µM) and EthD-1 (4 µM) for 30 min at room temperature in the dark.

Fluorescence images were acquired using an inverted fluorescence microscope equipped with FITC and TRITC filter sets (Olympus Corporation, Tokyo, Japan) at 10× magnification. Images were obtained at the spheroid mid-plane using a single focal plane to ensure consistent depth of analysis. No post-acquisition brightness or contrast adjustments were applied, allowing objective comparison between treatment groups.

### 2.11. Assessment of ROS-Mediated Cytotoxicity by NAC Pretreatment

To clarify the involvement of ROS in the cytotoxic response elicited by etoposide, curcumin, and their combined treatment, antioxidant rescue experiments were conducted using *N*-acetyl-*L*-cysteine (NAC). 3D tumor spheroids were generated according to the protocol described in Section 2.10 and cultured until compact, uniformly shaped spheroids were obtained.

Prior to drug exposure, mature spheroids were incubated with NAC (5 mM) for 1 h at 37 °C under standard culture conditions (5% CO_2_, humidified atmosphere). This concentration and pretreatment duration were selected based on widely used literature protocols demonstrating effective intracellular ROS scavenging without inducing cytotoxicity. Importantly, these conditions are commonly applied to assess the contribution of oxidative stress to drug-induced cytotoxicity rather than to achieve complete ROS suppression [23,24]. After NAC pretreatment, spheroids were treated with etoposide, curcumin, or their combination (etoposide + curcumin) using the same concentrations applied in earlier 3D spheroid experiments.

Untreated spheroids and spheroids exposed to NAC alone served as control groups. Following treatment, spheroids were incubated for the specified durations before subsequent analyses.

#### 2.11.1. Determination of Intracellular ROS Levels After NAC Pretreatment

Intracellular ROS production was evaluated using the fluorescent indicator 2′,7′-dichlorodihydrofluorescein diacetate (DCFH-DA). At the end of the treatment period, spheroids were dissociated into single-cell suspensions using Accutase (Sigma-Aldrich, St. Louis, MO, USA) to enable accurate quantification at the cellular level.

Cells were rinsed with PBS and incubated with DCFH-DA (10 µM) for 30 min at 37 °C in the dark. Excess probe was removed by washing with PBS, and fluorescence intensity was immediately measured by flow cytometry using a BD FACSVerse system (BD Biosciences, San Jose, CA, USA). Intracellular ROS levels were expressed as mean fluorescence intensity (MFI) and normalized to untreated control samples for comparative analysis.

#### 2.11.2. Evaluation of Cell Viability and Apoptosis Following NAC Pretreatment

The effect of NAC pretreatment on cell viability was evaluated using the CellTiter-Glo^®^ 3D Cell Viability Assay (Promega, Madison, WI, USA) according to the manufacturer’s instructions. Following treatment, luminescent signals proportional to intracellular ATP content were measured using a microplate reader. Cell viability was expressed as a percentage relative to untreated control spheroids and used to assess the extent to which NAC pretreatment attenuated etoposide- and curcumin-induced cytotoxicity.

### 2.12. Statistical Analysis

All experiments were performed with at least three independent biological replicates, and data are presented as mean ± standard error of the mean (SEM). Statistical analyses were conducted using SPSS software (version 25.0; IBM, USA). Normality of data distribution was assessed prior to parametric testing. For comparisons involving more than two treatment groups, one-way analysis of variance (ANOVA) was used to evaluate overall differences. When the primary interest was comparison of treatment groups relative to the vehicle-treated control (e.g., apoptosis, caspase activity, cytokine secretion, and ROS assays), Dunnett’s post hoc test was applied to control for multiple comparisons against a single reference group. In analyses where all pairwise group comparisons were relevant, Tukey’s post hoc test was used to control the family-wise error rate. The specific post hoc test applied for each experiment is indicated in the corresponding figure legends. A *p*-value < 0.05 was considered statistically significant. Quantitative drug–drug interaction analyses (Chou–Talalay CI and Bliss independence models) were performed using model-based calculations rather than inferential statistics, as described in Section 2.

## 3. Results

### 3.1. Baseline Etoposide Sensitivity of OVCAR-3 and SKOV-3 Cells

To evaluate the baseline responsiveness of ovarian cancer cell models to etoposide, OVCAR-3 and SKOV-3 cells were treated with increasing concentrations of etoposide (10–100 µM) for 48 h, and cell viability was assessed using the MTT assay. Etoposide exposure resulted in a clear, concentration-dependent reduction in cell viability in both cell lines (Figure S1).

Across all tested concentrations, OVCAR-3 cells exhibited a markedly greater reduction in viability compared with SKOV-3 cells. At 25 µM etoposide, OVCAR-3 cell viability decreased to approximately 40% of control levels, whereas SKOV-3 cells retained nearly 70% viability. This divergence became more pronounced at higher concentrations, with OVCAR-3 viability falling below 25% at 50 µM and approximately 10% at 100 µM, while SKOV-3 cells maintained significantly higher survival at corresponding doses.

Nonlinear regression analysis of the dose–response curves demonstrated that OVCAR-3 cells displayed a substantially lower IC_50_ value than SKOV-3 cells, indicating greater sensitivity to etoposide-induced cytotoxicity. In contrast, SKOV-3 cells showed a comparatively attenuated response, consistent with a comparatively attenuated response to etoposide.

**Figure 1:**
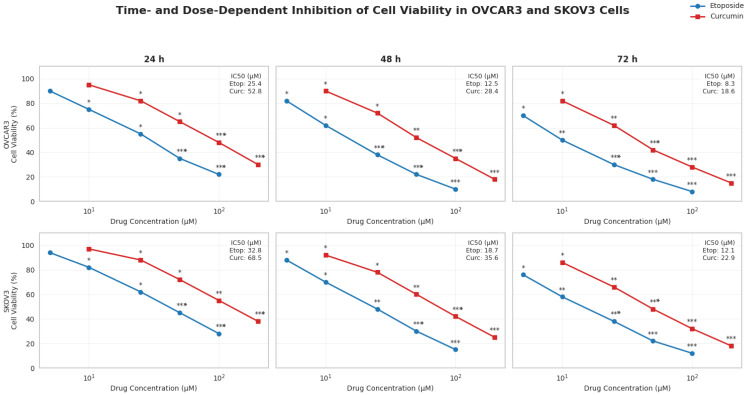
Time- and dose-dependent effects of etoposide and curcumin on ovarian cancer cell viability. OVCAR3 and SKOV3 ovarian cancer cells were treated with increasing concentrations of etoposide (5–100 µM) or curcumin (10–200 µM) for 24, 48, and 72 h. Cell viability was assessed using the MTT assay and expressed as the percentage of viable cells relative to untreated control cells. Data are presented as mean ± SEM from at least three independent experiments. Statistical significance was evaluated using one-way ANOVA followed by Dunnett’s post hoc test for multiple comparisons versus control. * *p* < 0.05, ** *p* < 0.01, *** *p* < 0.001 compared with control.

Collectively, these results confirm intrinsic differences in etoposide responsiveness between OVCAR-3 and SKOV-3 cells and support their use as complementary models representing relatively etoposide-sensitive and less-responsive ovarian cancer contexts, respectively (Figure 1).

### 3.2. Time- and Dose-Dependent Effects of Etoposide and Curcumin on Cell Viability

Cell viability was evaluated using the MTT assay following 24, 48, and 72 h of treatment with etoposide or curcumin in OVCAR3 and SKOV3 ovarian cancer cell lines. Both compounds induced a clear time- and dose-dependent reduction in cell viability in both cell models (Figure 1).

In OVCAR3 cells, IC_50_ values for etoposide were 25.4 ± 2.1 µM at 24 h, 12.5 ± 1.8 µM at 48 h, and 8.3 ± 1.2 µM at 72 h. Corresponding IC_50_ values for curcumin were 52.8 ± 4.5 µM, 28.4 ± 3.2 µM, and 18.6 ± 2.4 µM at 24, 48, and 72 h, respectively. At the 48 h time point, etoposide exhibited lower IC_50_ values than curcumin in OVCAR3 cells (Figure 1).

In SKOV3 cells, etoposide treatment resulted in IC_50_ values of 32.8 ± 3.0 µM at 24 h, 18.7 ± 2.1 µM at 48 h, and 12.1 ± 1.5 µM at 72 h. Curcumin IC_50_ values in SKOV3 cells were 68.5 ± 5.8 µM, 35.6 ± 4.1 µM, and 22.9 ± 2.8 µM at the respective time points. Compared with OVCAR3 cells, SKOV3 cells displayed higher IC_50_ values for both agents across all treatment durations.

For both compounds and cell lines, IC_50_ values decreased progressively with increasing exposure time. Based on these findings, the 48 h time point was selected for subsequent combination and mechanistic analyses (Figure 1).

### 3.3. Quantitative Assessment of Drug Synergy by Chou–Talalay Analysis

The interaction between etoposide and curcumin was quantitatively evaluated using the Chou–Talalay method under fixed-ratio combination conditions. Based on IC_50_ values obtained from single-agent treatments, etoposide and curcumin were combined at constant ratios of 1:2.3 in OVCAR3 cells and 1:1.9 in SKOV3 cells. Under these conditions, the combination treatment produced greater growth inhibition compared with either agent administered alone in both cell lines.

**Figure 2:**
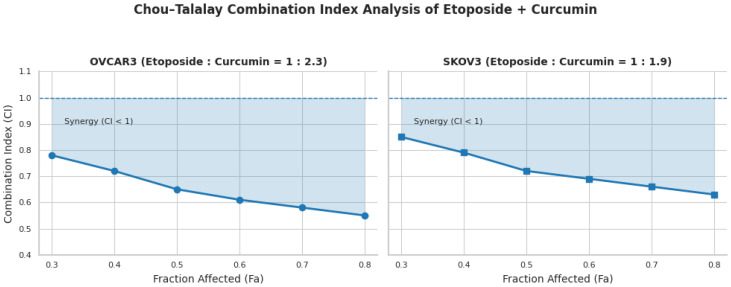
Chou–Talalay analysis of the combined effects of etoposide and curcumin in ovarian cancer cells. Dose–effect relationships of etoposide and curcumin were evaluated as single agents and in fixed-ratio combinations using the Chou–Talalay method. CI values were calculated over a wide range of fractional effects (Fa = 0.3–0.8). CI values < 1 indicate synergistic interactions, CI = 1 indicates additive effects, and CI values > 1 indicate antagonism. CI analysis was based on the median-effect model and did not involve inferential statistical testing. The combination treatment consistently yielded CI values below 1 in both OVCAR3 and SKOV3 cells, with mean CI values of 0.65 and 0.72, respectively. Data represent the mean of three independent experiments performed in triplicate.

CI values calculated across a broad range of fractional effects (Fa = 0.3–0.8) were consistently below 1 in both OVCAR3 and SKOV3 cells, indicating synergistic interactions throughout this effect range. The mean CI value was 0.65 for OVCAR3 cells and 0.72 for SKOV3 cells (Figure 2).

### 3.4. Dose–Response Interaction Patterns Revealed by Bliss Independence Analysis

**Figure 3:**
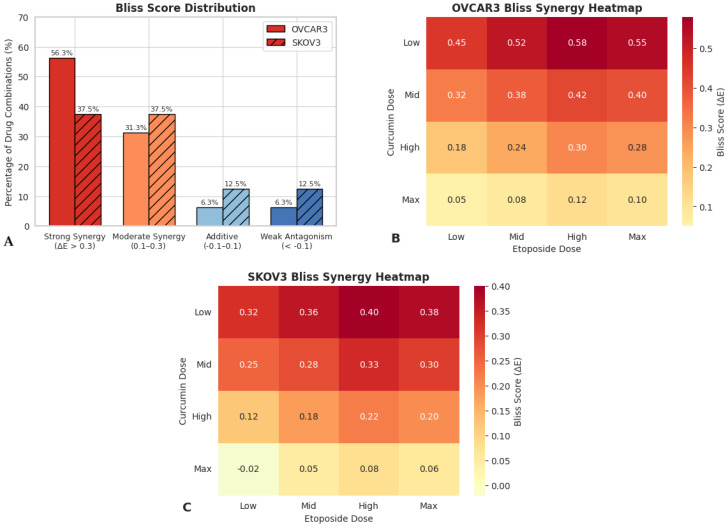
Bliss independence analysis of etoposide–curcumin combinations in ovarian cancer cells. (**A**) Distribution of Bliss synergy scores (ΔE) for etoposide and curcumin combinations in OVCAR3 and SKOV3 cells. Drug interactions were categorized as strong synergy (ΔE > 0.3), moderate synergy (0.1 < ΔE ≤ 0.3), additive interaction (−0.1 ≤ ΔE ≤ 0.1), or weak antagonism (ΔE < −0.1). Bars represent the percentage of tested dose combinations falling into each interaction category for the indicated cell lines. (**B**) Bliss independence heatmap illustrating dose-dependent interaction profiles between etoposide and curcumin in OVCAR3 cells. Positive ΔE values indicate synergistic interactions, whereas values close to zero or negative reflect additive or antagonistic effects. (**C**) Bliss independence heatmap showing interaction patterns in SKOV3 cells, displaying a generally similar but less pronounced distribution of synergistic interactions compared with OVCAR3 cells. Data are presented as mean values derived from three independent experiments performed in triplicate. Bliss independence analysis is a model-based, descriptive interaction assessment and does not involve inferential statistical testing. ΔE values were interpreted in a dose- and cell line–specific manner rather than as uniform synergy across all combinations. In particular, while OVCAR3 cells exhibited predominantly positive ΔE values across a broad concentration matrix, SKOV3 cells showed a more moderate and dose-restricted synergistic response, with attenuation or loss of synergy at higher curcumin dose levels.

Combination effects of etoposide and curcumin were further evaluated using the Bliss independence model. Heatmap visualization of Bliss synergy scores (ΔE) revealed predominantly positive values across multiple dose combinations, whereas additive interaction patterns were mainly observed at lower concentration ranges (Figure 3A).

In OVCAR3 cells, positive Bliss scores (ΔE > 0) were detected in 14 out of 16 tested dose combinations, corresponding to 87.5% of all combinations analyzed (Figure 3B). Bliss scores in this cell line ranged from −0.05 to 0.52. In SKOV3 cells, positive Bliss scores were observed in 12 out of 16 dose combinations (75%), with ΔE values ranging from −0.08 to 0.45 (Figure 3C).

Correlation analysis revealed a strong negative association between Bliss synergy scores and Chou–Talalay CI values in both cell lines (OVCAR3: r = −0.89, *p* < 0.001; SKOV3: r = −0.85, *p* < 0.001), indicating concordance between the two independent quantitative models used to evaluate drug–drug interactions.

### 3.5. Induction of Apoptotic Cell Death by Etoposide and Curcumin Treatment

**Figure 4:**
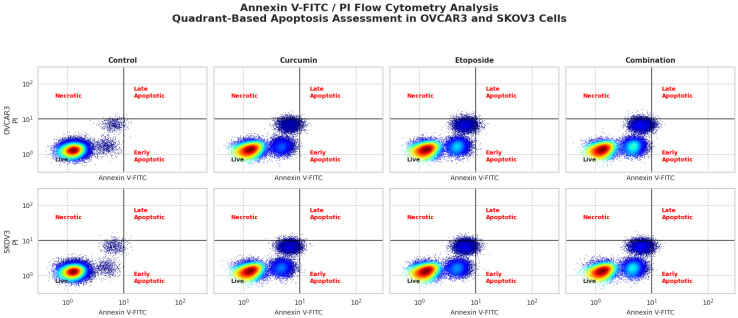
Flow cytometric analysis of apoptosis induced by etoposide and curcumin in ovarian cancer cells. Apoptotic cell death was assessed by Annexin V–FITC/PI double staining followed by flow cytometric analysis in OVCAR3 and SKOV3 cells. Representative four-quadrant dot plots illustrate the distribution of viable (Annexin V^−^/PI^−^), early apoptotic (Annexin V^+^/PI^−^), late apoptotic (Annexin V^+^/PI^+^), and necrotic (Annexin V^−^/PI^+^) cell populations after 48 h of treatment with curcumin (IC_50_), etoposide (IC_50_), or their combination. Data are presented as mean ± SEM from at least three independent experiments. Statistical significance was evaluated using one-way ANOVA followed by Dunnett’s post hoc test for comparisons versus control.

Apoptotic cell death was evaluated by flow cytometric analysis using Annexin V–FITC and PI double staining following 48 h of treatment in OVCAR3 and SKOV3 ovarian cancer cells (Figure 4). Both etoposide and curcumin, administered individually or in combination, increased the proportion of apoptotic cells compared with untreated controls.

In control conditions, the total apoptotic cell fraction was 6.2% in OVCAR3 cells and 5.8% in SKOV3 cells, with the majority of cells remaining viable (Annexin V^−^/PI^−^). In OVCAR3 cells, treatment with curcumin at its IC_50_ concentration increased the proportion of early apoptotic cells to 26.4% and late apoptotic/necrotic cells to 20.6%. Etoposide treatment resulted in early and late apoptotic/necrotic cell fractions of 32.3% and 26.1%, respectively.

A comparable pattern was observed in SKOV3 cells. Curcumin treatment increased early apoptotic cells to 26.8% and late apoptotic/necrotic cells to 26.2%, whereas etoposide treatment increased these populations to 27.6% and 29.1%, respectively.

Combined treatment with etoposide and curcumin at IC_50_ concentrations resulted in a marked increase in apoptotic cell populations in both cell lines. The total apoptotic fraction reached 64.5% in OVCAR3 cells and 62.7% in SKOV3 cells, representing a statistically significant increase compared with control and single-agent treatment groups (*p* < 0.001). Quadrant-based analysis demonstrated an increase in both early and late apoptotic populations accompanied by a reduction in viable cells (Figure 4). Detailed quadrant-based analysis of replicate Annexin V/PI flow cytometry data revealed that, in OVCAR3 cells, combination treatment resulted in approximately 18.2% early apoptotic (Annexin V^+^/PI^−^) and 46.3% late apoptotic/necrotic (Annexin V^+^/PI^+^) cells, together accounting for the observed total apoptotic fraction and accompanied by a marked reduction in the viable cell population. In SKOV3 cells, combination treatment similarly increased early apoptotic cells to approximately 21.7% and late apoptotic/necrotic cells to 41.0%. These quadrant-level distributions confirm that the enhanced apoptotic response induced by the etoposide–curcumin combination reflects coordinated activation of both early and late stages of apoptosis rather than nonspecific necrotic cell death.

### 3.6. Effects of Etoposide and Curcumin on Cell Cycle Distribution

**Figure 5:**
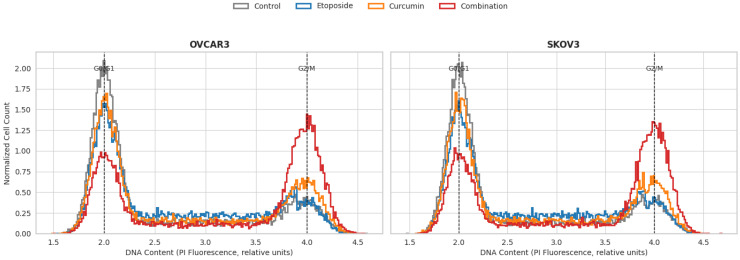
Cell cycle distribution in OVCAR3 and SKOV3 cells was analyzed by flow cytometry following PI staining after 48 h of treatment with etoposide, curcumin, or their combination. Representative DNA content histograms illustrate the relative distribution of cells in the G0/G1, S, and G2/M phases under each treatment condition. Combination treatment was associated with a pronounced accumulation of cells in the G2/M phase in both cell lines compared with control and single-agent treatments.

Cell cycle distribution was analyzed by flow cytometry following 48 h of treatment in OVCAR3 and SKOV3 ovarian cancer cells (Figure 5). Treatment with etoposide resulted in an increased proportion of cells in the S phase, whereas curcumin treatment led to a modest increase in the G2/M phase population in both cell lines.

Combined treatment with etoposide and curcumin produced a more pronounced accumulation of cells in the G2/M phase compared with either single-agent treatment in both OVCAR3 and SKOV3 cells. This increase in the G2/M phase cell fraction was statistically significant when compared with control and single-agent groups (*p* < 0.01) (Figure 5). Quantitative cell cycle analysis revealed that combination treatment increased the G2/M cell population to 42.5% in OVCAR3 cells and 38.7% in SKOV3 cells, compared with 19.1% and 18.4% in control cells, respectively (* *p* < 0.01). Single-agent treatments induced more modest increases that did not reach the levels observed with the combination.

### 3.7. Caspase-8/9 Activity Following Treatment

**Figure 6:**
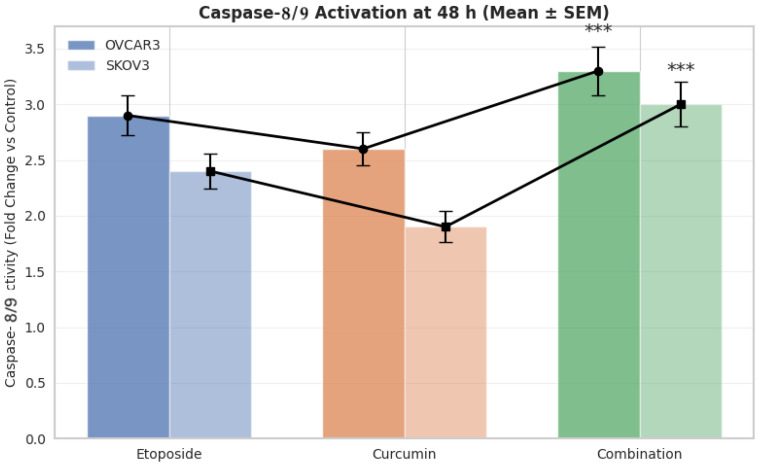
Caspase-8/9 activity following etoposide and curcumin treatment in ovarian cancer cells. Caspase-8 and caspase-9 activities were measured separately in OVCAR3 and SKOV3 cells after 48 h of treatment with etoposide (IC_50_), curcumin (IC_50_), or their fixed-ratio combination. As both caspases exhibited comparable activation patterns across treatment groups, their activities are presented together for clarity. Caspase activity is expressed as fold change relative to untreated control cells. Data are presented as mean ± SEM from at least three independent experiments. Statistical significance was evaluated using one-way ANOVA followed by Dunnett’s post hoc test. *** *p* < 0.001 compared with control.

Caspase-8 and caspase-9 activities were measured separately after 48 h of treatment in OVCAR3 and SKOV3 ovarian cancer cell lines (Figure 6). One-way ANOVA revealed statistically significant differences among treatment groups in both cell lines (OVCAR3: *p* < 0.001; SKOV3: *p* < 0.001). Both caspase-8 and caspase-9 exhibited comparable activation patterns across treatment groups; therefore, their activities are presented together for clarity.

In OVCAR3 cells, treatment with etoposide and curcumin alone increased caspase-8/9 activity by 2.9-fold and 2.6-fold, respectively, compared with control cells (*p* < 0.01). Combined treatment further increased caspase-8/9 activity to 3.3-fold relative to control, which was significantly higher than the activity observed with either single-agent treatment (*p* < 0.01).

In SKOV3 cells, etoposide and curcumin treatments increased caspase-8/9 activity by 2.4-fold and 1.9-fold, respectively, compared with control cells (*p* < 0.05). Combination treatment resulted in a 3.0-fold increase in caspase-8/9 activity, which was significantly greater than that observed following single-agent treatments (*p* < 0.01). Although combination treatment significantly increased caspase-8/9 activity compared with control conditions, the magnitude of increase relative to single-agent treatments was modest, indicating enhancement rather than a disproportionate amplification of caspase activation.

### 3.8. Effects of Etoposide and Curcumin on Cytokine Secretion

**Figure 7:**
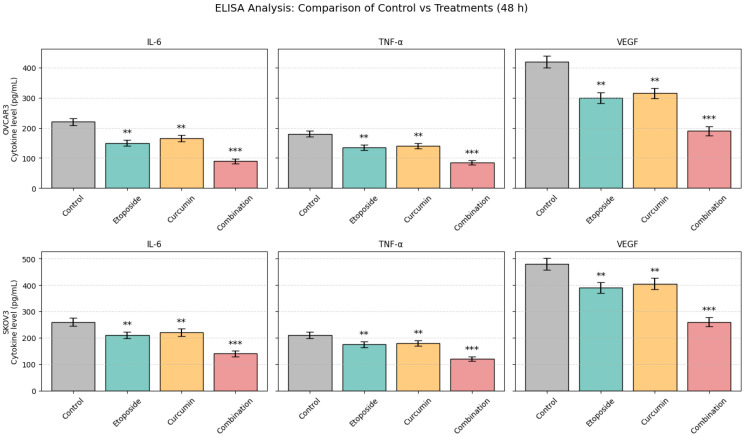
ELISA-based quantification of cytokine secretion following etoposide and curcumin treatment in ovarian cancer cells. OVCAR3 and SKOV3 cells were treated for 48 h with etoposide (IC_50_), curcumin (IC_50_), or their fixed-ratio combination, after which culture supernatants were collected for ELISA analysis. Secreted levels of IL-6, TNF-α, and VEGF were quantified and expressed as mean ± SEM from three independent experiments. Statistical significance was evaluated using one-way ANOVA followed by Dunnett’s post hoc test for comparisons versus the vehicle-treated control group. ** *p* < 0.01, *** *p* < 0.001 compared with control. No direct statistical comparisons between combination and single-agent treatments were performed.

The levels of IL-6, TNF-α, and VEGF in culture supernatants of OVCAR3 and SKOV3 cells were quantified by ELISA following 48 h of treatment (Figure 7). Cytokine concentrations differed significantly among treatment groups in both cell lines.

In OVCAR3 cells, treatment with etoposide or curcumin alone resulted in a moderate reduction in IL-6, TNF-α, and VEGF levels compared with control cells. Combined treatment produced a more pronounced decrease in IL-6 and TNF-α levels, which was statistically significant compared with control cells (*p* < 0.01) and more pronounced relative to single-agent treatments (*p* < 0.01). VEGF levels were also reduced to a greater extent in the combination group relative to single-agent treatments.

In SKOV3 cells, baseline cytokine levels were higher than those observed in OVCAR3 cells. Treatment with etoposide or curcumin alone led to limited reductions in IL-6 and TNF-α levels. In contrast, combined treatment resulted in a statistically significant decrease in IL-6, TNF-α, and VEGF levels compared with control cells (*p* < 0.05) (Figure 7).

### 3.9. Apoptotic and Microenvironment-Related Gene Expression Profiles

#### 3.9.1. Gene Expression Changes in OVCAR3 Cells

**Figure 8:**
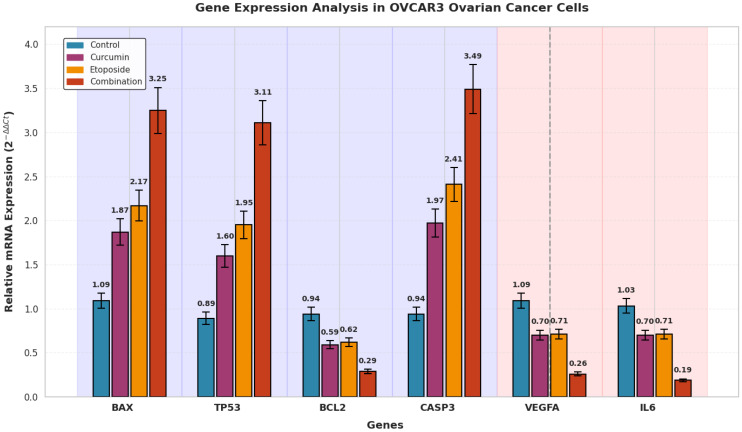
qRT-PCR analysis of apoptotic and microenvironment-related gene expression exclusively in OVCAR3 ovarian cancer cells. Relative mRNA expression levels of apoptosis-related genes (BAX, BCL2, TP53, CASP3) and microenvironment-associated genes (VEGFA, IL-6) were determined by quantitative real-time PCR in OVCAR3 ovarian cancer cells following treatment with etoposide, curcumin, or their combination and compared with untreated control samples. Gene expression levels are presented as fold change relative to control values. Data are expressed as mean ± SEM from at least three independent experiments Statistical significance was evaluated using one-way ANOVA followed by Dunnett’s post hoc test.

Gene expression analysis by qRT-PCR revealed significant alterations in apoptosis-related gene expression following combination treatment in OVCAR3 cells (Figure 8). Compared with control cells, the expression level of the pro-apoptotic gene BAX increased by 3.25-fold (*p* < 0.001), while TP53 expression was upregulated by 3.11-fold (*p* < 0.001). Similarly, CASP3 expression showed a 3.49-fold increase relative to control levels (*p* < 0.001).

In contrast, expression of the anti-apoptotic gene BCL2 was significantly reduced to 0.29-fold of control values, corresponding to a 3.26-fold downregulation (*p* < 0.001). As a result, the BCL2/BAX expression ratio decreased from 0.86 in control cells to 0.09 in the combination-treated group (Figure 8).

#### 3.9.2. Gene Expression Changes in SKOV3 Cells

**Figure 9:**
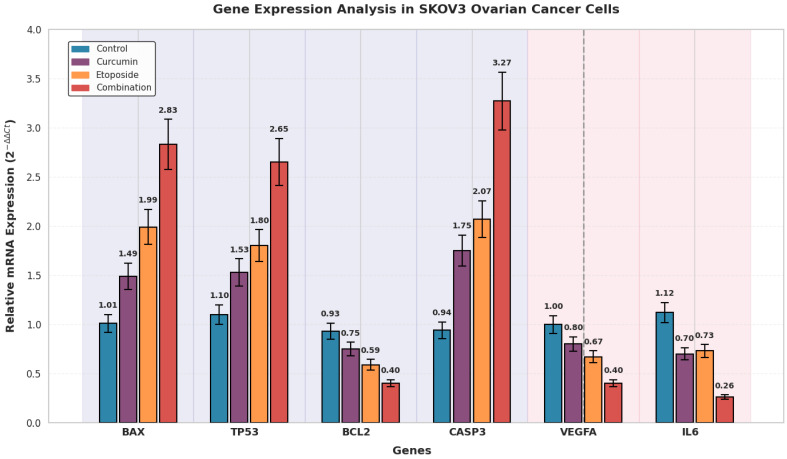
qRT-PCR analysis of apoptotic and microenvironment-related gene expression exclusively in SKOV3 ovarian cancer cells. Relative mRNA expression levels of apoptosis-related genes (BAX, BCL2, TP53, CASP3) and microenvironment-associated genes (VEGFA, IL-6) were determined by quantitative real-time PCR in SKOV3 ovarian cancer cells following treatment with etoposide, curcumin, or their combination and compared with untreated control samples. Gene expression levels are presented as fold change relative to control values. Data are expressed as mean ± SEM from at least three independent experiments. Statistical significance was evaluated using one-way ANOVA followed by Dunnett’s post hoc test.

Quantitative real-time PCR analysis demonstrated significant alterations in apoptosis-related gene expression following combination treatment in SKOV3 cells (Figure 9). Compared with control cells, BAX expression increased by 2.83-fold (*p* < 0.01), while TP53 expression was upregulated by 2.65-fold relative to control levels (*p* < 0.01). The expression of CASP3 was markedly increased by 3.27-fold compared with control cells (*p* < 0.001).

In contrast, BCL2 expression was significantly reduced to 0.40-fold of control values, corresponding to a 2.30-fold downregulation (*p* < 0.01) (Figure 9).

#### 3.9.3. Microenvironment-Related Cytokine Gene Expression

Quantitative real-time PCR analysis revealed significant changes in the expression of genes associated with angiogenesis and inflammation following combination treatment in both ovarian cancer cell lines (Figure 8 and Figure 9).

In OVCAR3 cells, Vascular Endothelial Growth Factor A (VEGFA) expression was reduced to 0.26-fold of control levels, corresponding to a 4.18-fold downregulation (*p* < 0.001) (Figure 8). In SKOV3 cells, VEGFA expression decreased to 0.40-fold of control values, representing a 2.50-fold reduction (*p* < 0.01) (Figure 9).

Similarly, IL-6 expression was markedly downregulated in both cell lines. In OVCAR3 cells, IL-6 expression decreased to 0.19-fold of control levels, corresponding to a 5.34-fold reduction (*p* < 0.001) (Figure 8). In SKOV3 cells, IL-6 expression was reduced to 0.26-fold of control values, representing a 4.27-fold decrease (*p* < 0.001) (Figure 9).

**Figure 10:**
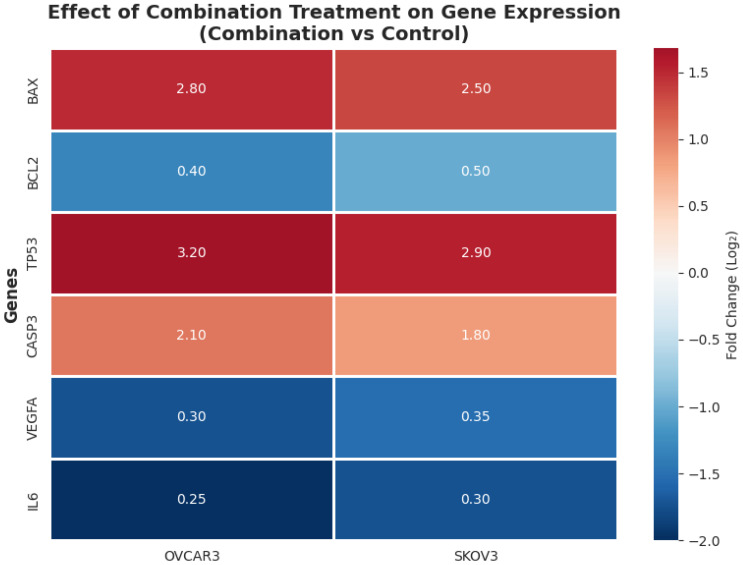
Heatmap representation of gene expression changes following combination treatment in ovarian cancer cells. Heatmap illustrating relative mRNA expression changes induced by the combined etoposide and curcumin treatment compared with untreated control conditions in OVCAR3 and SKOV3 ovarian cancer cells. The expression levels of apoptosis-related genes (BAX, TP53, CASP3, BCL2) and microenvironment-associated genes (VEGFA, IL-6) are shown as fold change values relative to control for each cell line. Red color intensity indicates upregulation, whereas blue color intensity indicates downregulation of gene expression following combination treatment.

Combination treatment resulted in a significant reduction in the expression levels of pro-angiogenic and pro-inflammatory genes compared with control and single-agent treatments (Figure 10).

#### 3.9.4. In Silico Bioinformatic Analyses and Protein–Protein Interaction Network

In silico bioinformatic analyses were performed to provide complementary, hypothesis-generating context for the experimental findings and to explore potential molecular targets and signaling nodes that may be relevant to the observed combination effects. Pharmacokinetic and drug-likeness prediction analyses indicated that both etoposide and curcumin complied with Lipinski’s rule of five and were predicted to exhibit high gastrointestinal absorption, supporting their suitability for further preclinical investigation.

Target prediction analysis using the SwissTargetPrediction platform (http://www.swisstargetprediction.ch; accessed on 10 January 2026) identified 43 high-probability protein targets shared by both compounds. These predicted targets were not experimentally validated in the present study and should therefore be interpreted as putative rather than confirmed molecular interactors. Protein–protein interaction (PPI) network analysis of this common target set was conducted using the STRING database. The resulting PPI network demonstrated a statistically significant interaction enrichment (PPI enrichment *p*-value < 1.0 × 10^−16^), indicating a non-random and highly interconnected network structure.

**Figure 11:**
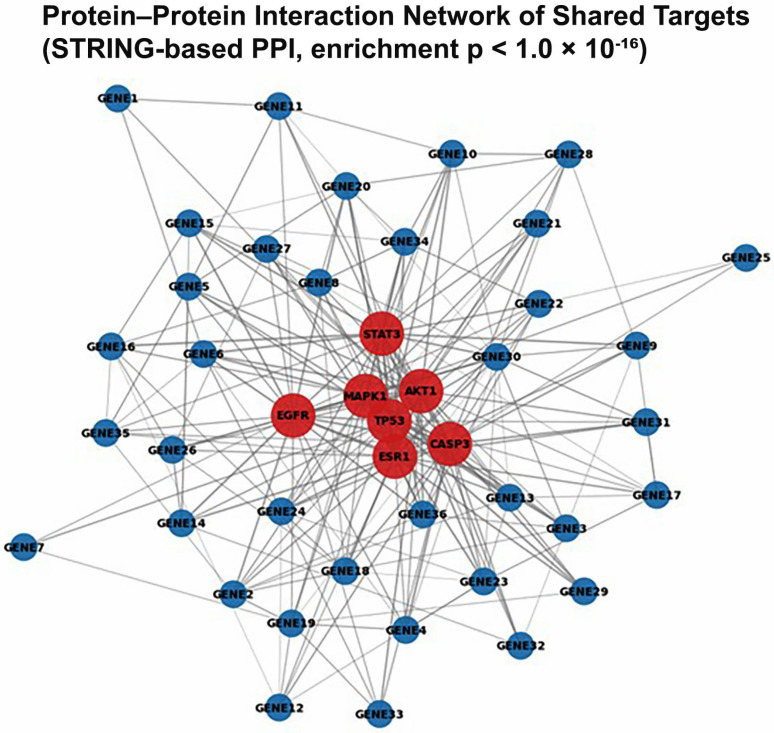
A STRING database–derived protein–protein interaction network constructed from the shared predicted targets of etoposide and curcumin, showing a highly interconnected network with significant enrichment (PPI enrichment *p* < 1.0 × 10^−16^). Hub genes identified by topological analysis, including p53, AKT1, CASP3, MAPK1, EGFR, STAT3, and ESR1, are highlighted in red and displayed with larger node sizes, reflecting their high degree of connectivity within the predicted network rather than confirmed functional dominance. Blue nodes represent other interacting proteins, while edges denote protein–protein interactions. This network provides a systems-level, hypothesis-generating overview of molecular pathways potentially relevant to apoptosis, cell cycle regulation, and cancer-related processes, without implying direct experimental validation or causal involvement.

Topological analysis of the PPI network identified several proteins, including p53, AKT1, CASP3, MAPK1, EGFR, STAT3, and ESR1, as nodes with high centrality values (Figure 11). The identification of these hub nodes reflects their prominent network connectivity within the predicted target set and does not imply direct causal involvement in the experimentally observed synergistic effects. Rather, these findings serve to highlight signaling pathways and molecular processes that may warrant further experimental validation in future mechanistic studies.

#### 3.9.5. GO and KEGG Enrichment Results of the Predicted Common Target Genes

**Figure 12:**
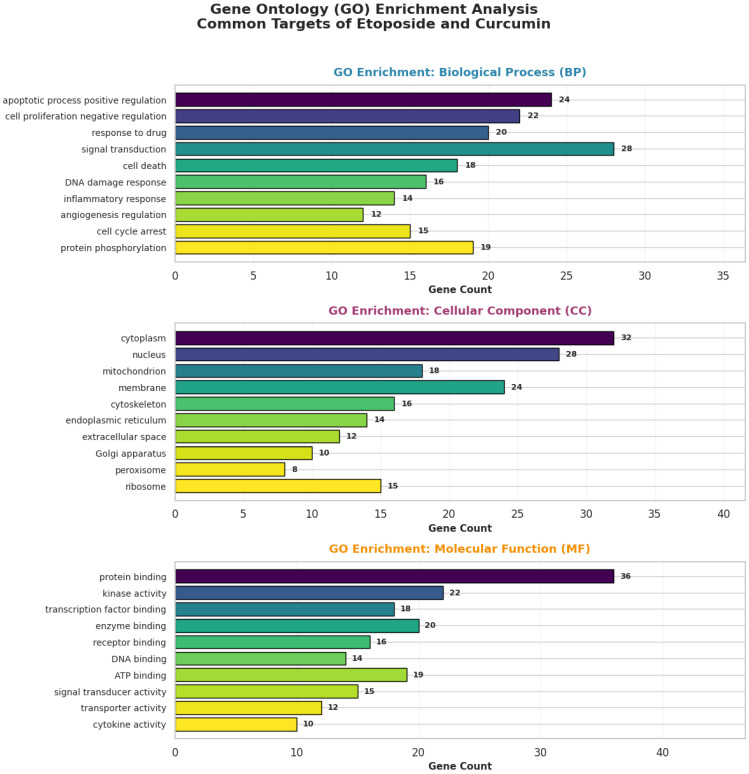
GO enrichment analysis of common predicted target genes of etoposide and curcumin. GO enrichment analysis was performed on the shared predicted target gene set of etoposide and curcumin to identify statistically overrepresented functional categories rather than experimentally validated pathway activation. Bar plots illustrate the most significantly enriched terms in BP, CC, and MF categories based on FDR-adjusted *p*-values (< 0.05). Enriched biological processes include positive regulation of apoptotic process, negative regulation of cell proliferation, response to drug, and cell cycle-related processes. Cellular component analysis revealed enrichment in cytoplasm, nucleus, mitochondrion, and membrane-related compartments, while molecular function analysis highlighted protein binding, kinase activity, transcription factor binding, and enzyme binding. The x-axis represents the number of genes associated with each GO term. These enrichment results reflect statistical overrepresentation within a computationally predicted target set and should therefore be interpreted as hypothesis-generating rather than as direct mechanistic evidence of pathway modulation.

Functional enrichment analysis of the predicted common target gene set was performed using GO and KEGG databases to explore potential biological processes and pathways that may be associated with the observed experimental effects (Figure 12). In the GO Biological Process (BP) category, the target genes were enriched in terms related to processes commonly implicated in cancer biology, including positive regulation of apoptotic process, negative regulation of cell proliferation, and response to drug.

KEGG pathway enrichment analysis further indicated that the predicted target genes were associated with several signaling pathways, including the p53 signaling pathway, PI3K–Akt signaling pathway, apoptosis, and HIF-1 signaling pathway (Figure 12). These enriched pathways overlap with signaling axes frequently involved in cellular stress responses, survival regulation, and apoptosis, and are broadly consistent with the phenotypic changes observed experimentally. However, it should be emphasized that these enrichment results are derived solely from in silico predictions and do not demonstrate direct pathway activation or mechanistic causality. Enrichment significance was determined based on FDR-adjusted *p*-values (<0.05), and the findings should therefore be interpreted as hypothesis-generating and supportive rather than confirmatory.

### 3.10. Effects of Etoposide and Curcumin in a 3D Tumor Spheroid Model

Drug concentrations used in 3D spheroid experiments were adjusted relative to 2D cultures to account for diffusion limitations, as described in Section 2.10.2.

#### 3.10.1. Formation and Morphological Alterations of 3D Tumor Spheroids

**Figure 13:**
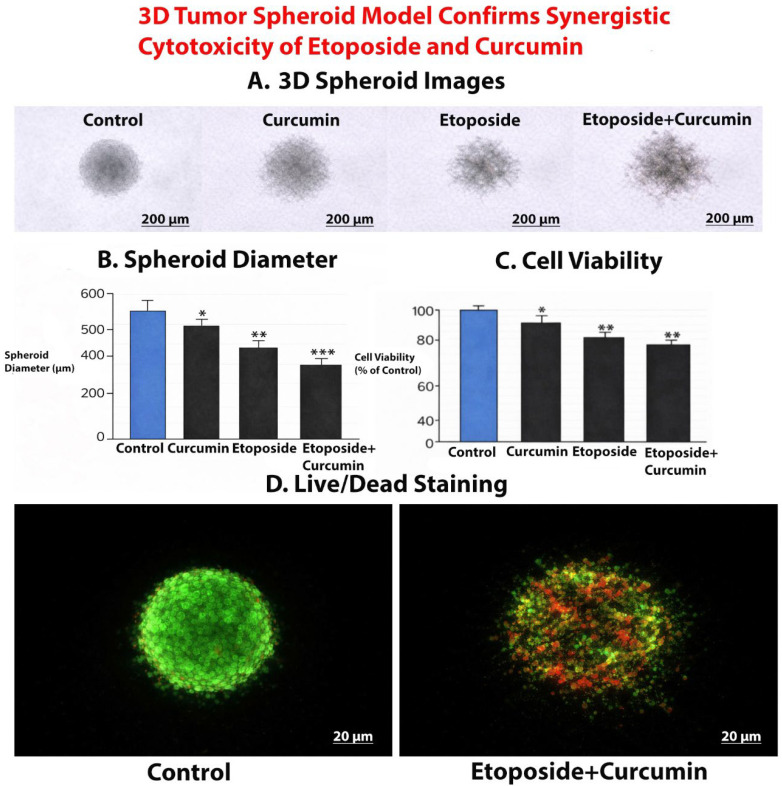
Effects of etoposide and curcumin on 3D tumor spheroids. (**A**) Representative bright-field images of 3D tumor spheroids formed from ovarian cancer cells under control conditions and following treatment with curcumin, etoposide, or their combination (etoposide + curcumin). Control spheroids display a compact, spherical morphology with smooth borders. Curcumin treatment results in a moderate reduction in spheroid size, whereas etoposide treatment induces a more pronounced shrinkage accompanied by partial loss of compactness. Combined etoposide and curcumin treatment leads to marked spheroid shrinkage and disrupted structural integrity. Scale bar = 200 µm. (**B**) Quantitative analysis of spheroid diameter measured from bright-field images using ImageJ software. Data are presented as mean ± SEM from at least three independent biological experiments, with a minimum of three spheroids analyzed per condition. Treatment with curcumin or etoposide resulted in a significant reduction in spheroid diameter compared with control conditions, while the etoposide + curcumin combination was associated with the greatest reduction in spheroid size relative to control. Statistical significance was evaluated using one-way ANOVA followed by Dunnett’s post hoc test. * *p* < 0.05, ** *p* < 0.01, *** *p* < 0.001 compared with control. (**C**) Cell viability of 3D tumor spheroids assessed using the CellTiter-Glo® 3D Cell Viability Assay after 72 h of treatment. Viability values are expressed as a percentage relative to vehicle-treated control spheroids. Treatment with curcumin or etoposide resulted in a significant reduction in spheroid viability compared with control conditions, while the etoposide + curcumin combination was associated with a further decrease in cell viability relative to control. Data are presented as mean ± SEM from at least three independent experiments. Statistical significance was evaluated using one-way ANOVA followed by Dunnett’s post hoc test. * *p* < 0.05, ** *p* < 0.01 compared with control. (**D**) Representative live/dead fluorescence staining of 3D tumor spheroids following treatment. Live cells are indicated by green fluorescence (Calcein-AM), and dead cells by red fluorescence (Ethidium homodimer-1). Control spheroids exhibit predominantly green fluorescence, indicating high viability, whereas spheroids treated with the etoposide + curcumin combination show extensive red fluorescence and reduced green signal, reflecting widespread cell death and disruption of spheroid architecture. Scale bar = 20 µm.

Under control conditions, ovarian cancer cells formed compact, spherical, and well-defined 3D tumor spheroids with smooth and regular borders. Treatment with curcumin resulted in a moderate reduction in spheroid size while largely preserving spheroid integrity. Etoposide treatment induced a more pronounced decrease in spheroid size, accompanied by partial loss of spheroid compactness. Notably, combined etoposide and curcumin treatment led to marked spheroid shrinkage and substantial loss of structural integrity, characterized by irregular borders and fragmented morphology (Figure 13A).

#### 3.10.2. Quantitative Reduction in Spheroid Size Following Combination Treatment

Quantitative analysis of spheroid diameter revealed significant differences among treatment groups. Compared with control spheroids, both curcumin and etoposide treatments significantly reduced spheroid diameter. The most pronounced reduction was observed in the etoposide + curcumin group, which exhibited a significantly smaller spheroid diameter than either single-agent treatment (Figure 13B). These results demonstrate that the antiproliferative effects observed in 2D cultures are maintained and amplified in a 3D tumor spheroid model.

#### 3.10.3. Suppression of Cell Viability in 3D Tumor Spheroids

Cell viability analysis showed that curcumin and etoposide alone significantly reduced spheroid viability compared with control spheroids. Importantly, combined etoposide and curcumin treatment resulted in the strongest suppression of spheroid viability, significantly exceeding the effects of either agent alone (Figure 13C). These findings indicate that the synergistic cytotoxic interaction between etoposide and curcumin observed in monolayer cultures is preserved in the 3D spheroid context.

#### 3.10.4. Live/Dead Fluorescence Staining Reveals Enhanced Cell Death Following Combination Treatment

Live/dead fluorescence staining demonstrated that control spheroids predominantly exhibited green fluorescence, indicating a high proportion of viable cells. Spheroids treated with either curcumin or etoposide displayed increased red fluorescence, consistent with enhanced cell death. In contrast, spheroids treated with the etoposide + curcumin combination showed extensive red fluorescence accompanied by a marked reduction in green signal, reflecting widespread loss of cell viability and pronounced disruption of spheroid architecture (Figure 13D).

### 3.11. NAC Pretreatment Attenuates Etoposide- and Curcumin–Induced ROS Generation

**Figure 14:**
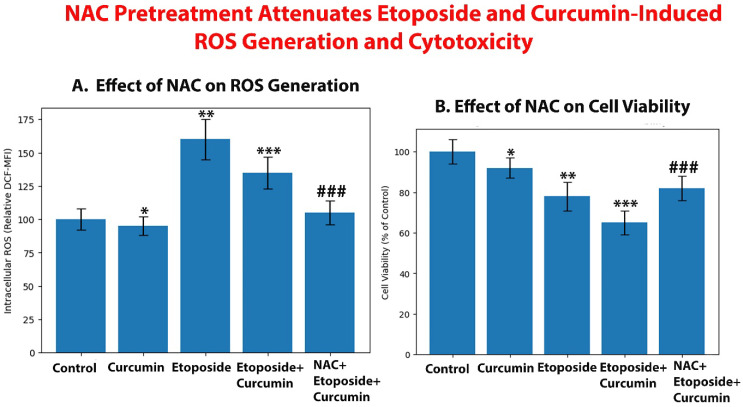
NAC pretreatment modulates etoposide- and curcumin–induced ROS generation and cytotoxicity in 3D tumor spheroids. (**A**) Intracellular ROS levels were quantified using the DCFH-DA assay and expressed as relative DCF MFI. Etoposide treatment significantly increased intracellular ROS levels compared with control spheroids, while the etoposide + curcumin combination also elevated ROS levels relative to control but to a lesser extent than etoposide alone, indicating partial modulation of etoposide-driven oxidative stress by curcumin. Pretreatment with NAC significantly reduced ROS accumulation in spheroids treated with the etoposide + curcumin combination. (**B**) Cell viability was assessed using an ATP-based assay and expressed as a percentage of vehicle-treated control spheroids. Etoposide and etoposide + curcumin treatments significantly reduced spheroid viability, with the greatest reduction observed in the combination group. NAC pretreatment partially restored cell viability in spheroids treated with the etoposide + curcumin combination without fully returning viability to control levels, indicating partial ROS-dependent cytotoxicity. Data are presented as mean ± SEM from at least three independent biological experiments. Statistical significance was evaluated using one-way ANOVA followed by Dunnett’s post hoc test. * *p* < 0.05, ** *p* < 0.01, *** *p* < 0.001 vs. control; ### *p* < 0.001 vs. etoposide + curcumin.

Treatment of 3D tumor spheroids with etoposide was associated with a pronounced increase in intracellular ROS levels compared with control spheroids, consistent with its established mechanism involving DNA damage-associated oxidative stress. Curcumin treatment alone did not increase intracellular ROS levels relative to control spheroids and instead was associated with lower ROS levels compared with etoposide-treated spheroids, consistent with its reported antioxidant properties. In spheroids treated with the etoposide + curcumin combination, intracellular ROS levels were elevated relative to control spheroids but remained lower than those observed following etoposide treatment alone, indicating that curcumin partially modulates etoposide-driven oxidative stress rather than acting as an independent ROS inducer. Importantly, pretreatment with the ROS scavenger NAC significantly reduced ROS accumulation in combination-treated spheroids, as indicated by decreased DCF fluorescence intensity. NAC treatment alone did not appreciably alter basal ROS levels compared with control spheroids (Figure 14A).

#### 3.11.1. ROS Scavenging Partially Reverses Etoposide + Curcumin–Induced Cytotoxicity

In parallel with the observed reduction in intracellular ROS levels, NAC pretreatment attenuated the cytotoxic effects induced by the etoposide + curcumin combination in 3D tumor spheroids. Spheroids pretreated with NAC exhibited higher cell viability compared with spheroids treated with the combination alone (Figure 14B). However, NAC pretreatment did not restore cell viability to control levels, indicating that suppression of etoposide-associated ROS provides partial but incomplete protection against combination-induced cytotoxicity.

#### 3.11.2. ROS Contributes to, but Does Not Fully Mediate, Etoposide + Curcumin–Induced Cell Death

Although NAC-mediated neutralization of ROS diminished oxidative stress and partially restored cell viability, the incomplete reversal of cytotoxicity suggests that ROS is an important contributing factor but not the sole driver of etoposide + curcumin–induced cell death. Taken together, these findings indicate that the enhanced cytotoxic effects of the combination are primarily driven by etoposide-associated oxidative stress, which is partially buffered by curcumin, while additional ROS-independent mechanisms are likely involved in mediating the overall cytotoxic response. This supports a multifactorial mode of action underlying the observed synergy in 3D tumor spheroids.

## 4. Discussion

This study provides quantitative and complementary evidence demonstrating a synergistic interaction between etoposide and curcumin in human ovarian cancer cell models. Using multiple independent analytical approaches, including Chou–Talalay and Bliss independence models, the combination consistently produced enhanced antiproliferative and pro-apoptotic effects compared with either agent alone. Beyond synergy quantification, the present findings extend existing combination therapy research by integrating transcriptional profiling, caspase pathway activation, tumor microenvironment-related cytokine modulation, and 3D tumor spheroid validation within a single experimental framework. Importantly, the observed synergy was accompanied by coordinated regulation of apoptosis-related genes, suppression of angiogenic and inflammatory mediators, and activation of both intrinsic and extrinsic apoptotic pathways. Validation of these effects in 3D spheroid cultures further supports the translational relevance of the combination, while ROS modulation experiments suggest that oxidative stress contributes to, but does not solely account for, the enhanced cytotoxic response. Collectively, these results position the etoposide–curcumin combination as a mechanistically grounded and multi-layered therapeutic strategy worthy of further preclinical investigation.

The rigorous quantification of drug–drug interactions using both Chou–Talalay and Bliss independence models represents a key methodological strength of the present study. The mean CI values of 0.65 in OVCAR3 cells and 0.72 in SKOV3 cells indicate a consistent synergistic interaction and compare favorably with previously reported etoposide-based combinations. For example, etoposide–cisplatin combinations in ovarian cancer models have typically yielded CI values ranging from 0.7 to 0.9 [25], placing the magnitude of synergy observed here within a lower and more consistent range. Importantly, Bliss independence analysis demonstrated synergistic interactions across a broad spectrum of dose combinations, with positive Bliss scores observed in 87.5% of tested conditions in OVCAR3 cells and 75% in SKOV3 cells within the examined concentration range (0.25× to 2× IC_50_). This distribution contrasts with many previously reported combination strategies in which synergy is restricted to narrow dose ratios or limited concentration windows [26]. The strong negative correlations observed between CI values and Bliss synergy scores (OVCAR3: r = −0.89; SKOV3: r = −0.85) further support the internal consistency of the interaction analyses and underscore the robustness of the quantitative framework employed, an aspect not uniformly addressed in combination therapy studies. Collectively, these quantitative metrics suggest that the etoposide–curcumin combination achieves enhanced cytotoxic efficacy relative to additive expectations across multiple dose levels. While these findings are derived from preclinical in vitro models, they raise the possibility that comparable biological effects might be attainable at reduced effective doses, a hypothesis that warrants further investigation in more advanced preclinical systems. Importantly, the magnitude and consistency of synergistic interactions differed between the two ovarian cancer cell models examined. In OVCAR3 cells, synergistic effects were robust and observed across a broad range of dose combinations and fractional effect levels, as consistently supported by both Chou–Talalay and Bliss independence analyses. In contrast, SKOV3 cells exhibited a more moderate and dose-restricted synergistic response, with attenuation or loss of synergy observed at higher curcumin concentrations. These findings indicate that the strength and stability of the etoposide–curcumin interaction are cell line-dependent and highlight the influence of intrinsic biological heterogeneity on combination treatment responses. Accordingly, synergistic efficacy was interpreted in a dose- and context-dependent manner rather than as a uniform interaction across all experimental conditions.

The extent of apoptotic induction observed with combination treatment in the present study (64.5% in OVCAR3 cells and 62.7% in SKOV3 cells) exceeds apoptotic rates commonly reported for single-agent etoposide or curcumin in comparable ovarian cancer models. Previous studies have typically described apoptotic fractions of approximately 30–40% for etoposide at IC_50_ concentrations [27] and 20–30% for curcumin administered alone [28], highlighting the enhanced magnitude of apoptosis achieved with the combination approach. Notably, the observed apoptotic response with combination treatment was greater than the level predicted by simple additive effects of the individual agents. In OVCAR3 cells, the expected arithmetic sum of apoptosis induced by single-agent treatments was approximately 52%, whereas the experimentally observed apoptotic fraction reached 64.5%. This quantitative discrepancy supports the presence of synergistic, rather than merely additive, apoptotic effects and is consistent with the interaction profiles obtained from Chou–Talalay and Bliss independence analyses.

The caspase activation profiles observed in this study provide mechanistic context for the enhanced apoptotic response elicited by the combination treatment. Concurrent increases in caspase-8 (4.95-fold) and caspase-9 (6.72-fold) activity indicate engagement of both extrinsic and intrinsic apoptotic pathways. Activation of multiple apoptotic initiation routes may be relevant, as dysregulation or selective suppression of individual apoptotic pathways has been implicated in cancer cell survival and therapy resistance [29]. Notably, the relative magnitude of caspase-9 activation compared with caspase-8 (caspase-9/caspase-8 ratio of approximately 1.36) suggests a stronger contribution of the mitochondrial pathway, while maintaining measurable activation of the death receptor-associated pathway. This pattern is consistent with a coordinated apoptotic response and may help explain the robust apoptotic induction observed following combination treatment. Although caspase-8 and caspase-9 activities were presented together due to their broadly similar activation trends across treatment groups, the relative magnitude of caspase activation provides additional mechanistic insight. Specifically, the higher fold increase observed for caspase-9 compared with caspase-8 suggests a more prominent contribution of the mitochondrial apoptotic pathway, while still indicating measurable engagement of death receptor-associated signaling. Importantly, this interpretation is based on relative activation levels rather than exclusive pathway attribution and does not imply functional independence of either pathway. Moreover, the absence of direct executioner caspase-3 activity measurements represents a limitation, and therefore conclusions regarding apoptotic execution should be interpreted with appropriate caution. Notably, although combination treatment consistently enhanced apoptotic cell death, the magnitude of caspase-8 and caspase-9 activation did not uniformly exceed the effects of etoposide alone across all experimental conditions. Therefore, caspase activation data should be interpreted as supportive of enhanced apoptosis rather than definitive evidence of molecular synergy at the level of apoptotic initiator caspases.

Cell cycle analysis demonstrated that combination treatment resulted in a more pronounced accumulation of cells in the G2/M phase compared with either single-agent treatment. In OVCAR3 cells, the proportion of G2/M-phase cells reached 42.5% following combination treatment, whereas etoposide and curcumin alone induced G2/M arrest in 21.3% and 23.0% of cells, respectively. This enhanced G2/M accumulation was statistically significant (*p* < 0.01) and was consistently observed across experiments. The magnitude of G2/M-phase enrichment with combination treatment exceeded that observed with single agents when expressed as a relative increase over control conditions (approximately 123% for the combination versus 112% for etoposide alone). Although this difference appears moderate in absolute terms, it reflects a measurable and statistically significant shift in cell cycle distribution. Given the established role of the G2/M checkpoint in regulating cellular responses to DNA damage, such modulation may contribute to the enhanced cytotoxic response observed with the combination treatment, particularly in the context of DNA-damaging agents such as etoposide [30]. It is important to note that G2/M phase accumulation may reflect distinct biological processes, including activation of the G2/M DNA damage checkpoint or progression toward mitotic catastrophe, which represent mechanistically different outcomes. While the present data clearly demonstrate enhanced G2/M accumulation following combination treatment, the experimental design does not allow discrimination between checkpoint-mediated cell cycle arrest and mitotic catastrophe. Given that etoposide is a DNA-damaging agent and that G2/M arrest is accompanied by robust apoptotic activation in this study, prolonged checkpoint activation may contribute to subsequent apoptotic cell death; however, further studies incorporating mitotic markers and high-resolution imaging would be required to delineate these mechanisms.

ELISA-based cytokine profiling demonstrated that combination treatment resulted in a more pronounced suppression of inflammatory and angiogenic mediators compared with single-agent treatments. In OVCAR3 cells, IL-6 levels were reduced to 19% of control values (corresponding to a 5.34-fold reduction), whereas curcumin and etoposide alone reduced IL-6 levels to approximately 70% and 68% of control, respectively. This corresponds to an approximately 3.5-fold greater degree of IL-6 suppression with the combination treatment. A similar trend was observed for VEGFA, where combination treatment reduced expression to 0.26-fold of control levels (4.18-fold reduction), exceeding the effects observed with single-agent treatments by approximately 2.5-fold. Elevated IL-6 and VEGFA levels have been associated with poor prognosis, angiogenesis, and chemotherapy resistance in ovarian cancer [31,32], placing the observed degree of cytokine suppression in a relevant biological context. Previous studies have reported IL-6 suppression in the range of 30–50% for curcumin monotherapy and 20–40% for conventional chemotherapeutic agents in comparable ovarian cancer models [33]. In this context, the magnitude of IL-6 reduction observed here (approximately 81%) represents a quantitatively greater effect. Moreover, the concurrent downregulation of IL-6, TNF-α, and VEGFA suggests a coordinated modulation of cytokine expression, whereas many reported combination strategies exhibit more selective cytokine effects. However, because cytokine levels were measured in culture supernatants without normalization to cell number or viability, the observed reductions may partially reflect decreased viable cell mass rather than direct anti-inflammatory synergy relative to etoposide alone. Accordingly, cytokine modulation findings should be interpreted as supportive of combination-associated effects rather than conclusive evidence of synergistic anti-inflammatory activity.

The observation that SKOV3 cells exhibited higher basal levels of inflammatory and angiogenic cytokines compared with OVCAR3 cells is consistent with previously reported intrinsic biological differences among ovarian cancer cell lines. Comprehensive genomic profiling studies have demonstrated pronounced molecular heterogeneity between commonly used ovarian cancer cell lines, with SKOV3 cells displaying genomic and transcriptional features that diverge from those of high-grade serous ovarian cancer and are associated with altered inflammatory and stress-response signaling profiles [34]. Such inherent differences may underlie elevated baseline cytokine production and contribute to distinct treatment response patterns. Furthermore, accumulating evidence indicates that a pre-existing inflammatory state within ovarian cancer cells and their associated microenvironment can critically influence chemotherapy responsiveness. Elevated basal expression of inflammatory mediators and activation of pathways such as NF-κB and STAT signaling have been linked to differential treatment sensitivity and adaptive survival responses in ovarian cancer [35]. In this context, the higher baseline cytokine levels observed in SKOV3 cells may reflect an intrinsically more inflammatory phenotype that modulates both basal signaling activity and treatment-induced cytokine suppression. In addition, interactions between ovarian cancer cells and components of the tumor microenvironment, including fibroblast-derived factors and TGF-β–regulated stromal signaling, have been shown to promote pro-inflammatory and pro-invasive phenotypes through NF-κB-dependent mechanisms [36]. Such microenvironment-associated signaling programs may further contribute to cell line–specific differences in cytokine expression and inflammatory tone, even under in vitro conditions. While the present study was not designed to dissect the molecular determinants underlying these baseline differences, acknowledging such intrinsic heterogeneity provides important biological context for interpreting cytokine modulation and treatment responses across distinct ovarian cancer cell models.

The qRT-PCR data reveal a coordinated transcriptional response that quantitatively supports the phenotypic findings observed in this study. In OVCAR3 cells, the BCL2/BAX expression ratio decreased from 0.86 in control cells to 0.09 following combination treatment, representing an order-of-magnitude shift. This degree of reduction exceeds the 2–4-fold changes typically reported for single-agent treatments in ovarian cancer models [37] and is consistent with a substantially lowered apoptotic threshold. The magnitude of gene expression modulation observed with the combination treatment, including a 3.25–3.49-fold upregulation of pro-apoptotic genes and a 3.26–5.34-fold downregulation of anti-apoptotic and cytokine-related genes, compares favorably with values reported for either etoposide or curcumin administered alone in similar experimental systems [38,39]. These transcriptional changes align with the enhanced apoptotic and antiproliferative responses observed at the cellular level. Notably, suppression of IL-6 expression reached 5.34-fold in OVCAR3 cells, exceeding the approximately 2-fold reductions commonly achieved with targeted IL-6 inhibition strategies in preclinical models [32]. While derived from in vitro systems, this degree of cytokine modulation suggests that the combined treatment may influence inflammatory signaling more broadly than single-pathway interventions.

The in silico analyses identified 43 common predicted targets for etoposide and curcumin, with a PPI enrichment *p*-value < 1.0 × 10^−16^, indicating a highly non-random and densely connected interaction network. Within this network, several genes with high centrality values were subsequently supported by experimental findings, including increased TP53 mRNA expression and CASP3 and downregulation of IL-6 at the transcriptional level. The concordance between predicted hub genes and experimentally observed expression changes supports the relevance of the network-based analytical framework employed in this study. In addition, KEGG pathway enrichment analysis revealed significant association of the predicted target set with apoptosis-related and p53 signaling pathways (*p* = 1.2 × 10^−15^), which aligns with the observed enhancement of apoptotic responses and increased TP53 expression in treated cells. Together, these findings indicate a consistency between the computational predictions and experimental outcomes. However, it should be emphasized that the molecular targets and signaling pathways identified through SwissTargetPrediction and network-based analyses are derived from computational predictions and were not experimentally validated in the present study. Therefore, these bioinformatics findings should be interpreted as supportive and hypothesis-generating rather than as direct mechanistic evidence. Importantly, interpretation of p53 pathway involvement should be made with caution in SKOV3 cells, which are widely reported to exhibit very low or undetectable p53 protein expression and impaired p53-dependent transcriptional activity. Accordingly, p53-related enrichment results in SKOV3 cells should be considered hypothesis-generating rather than indicative of functional p53 signaling, and future studies incorporating direct validation of TP53/p53 status and downstream functional readouts, such as p21 induction following DNA damage, would be required to substantiate these pathway-level inferences.

From a quantitative perspective, the etoposide–curcumin combination exhibits favorable performance when compared with several previously reported combination strategies. Etoposide–platinum-based combinations have generally been reported to induce approximately 1.5–2.0-fold increases in apoptotic activity relative to single-agent treatments [40], whereas the present combination achieved a 2.1–2.3-fold enhancement across the examined ovarian cancer cell models. Similarly, when compared with curcumin-based combinations involving other chemotherapeutic agents, which have shown variable or moderate synergy indices [41], the etoposide–curcumin combination demonstrated consistently lower CI values and broader synergy profiles across multiple dose levels. Importantly, the observed effects reflect concurrent modulation of multiple biological processes, including apoptotic signaling, cell cycle regulation, and cytokine expression. Such multi-level engagement may contribute to the quantitative differences observed relative to combinations that predominantly target a single pathway.

The IC_50_ values observed for the etoposide–curcumin combination fall within concentration ranges that have been reported as pharmacologically relevant in preclinical and translational studies. Based on the in vitro efficacy data, the estimated effective concentrations correspond to approximately 8–10 µM for etoposide and 18–22 µM for curcumin, which are consistent with exposure ranges described in previous experimental and pharmacokinetic reports [42,43]. The identification of a 48 h treatment duration as optimal for combination efficacy is in line with commonly used experimental timeframes in chemotherapy response studies. While these observations are derived from in vitro systems, they provide a practical framework for designing subsequent preclinical evaluations. In addition, the differential sensitivity observed between OVCAR3 and SKOV3 cells, with OVCAR3 cells exhibiting greater responsiveness, highlights the potential influence of underlying molecular characteristics on treatment outcomes. Such variability supports the rationale for further investigation into molecular determinants of response, which may inform stratification strategies in future preclinical or translational studies.

While the present study provides comprehensive and quantitative insights into the synergistic effects of the etoposide–curcumin combination, several limitations should be acknowledged. First, the experimental findings are based on in vitro cell culture models, which, although highly informative for mechanistic evaluation, cannot fully recapitulate the spatial, cellular, and biochemical complexity of the tumor microenvironment observed in vivo. Future studies incorporating advanced 3D culture systems, such as tumor spheroids and patient-derived organoids, would be valuable to further validate the translational relevance of the observed effects. In addition, in vivo pharmacokinetic and pharmacodynamic studies are required to determine whether the predicted drug-like properties and effective concentration ranges observed in vitro can be achieved and maintained under physiological conditions. Assessment of the effects of the combination treatment on non-malignant ovarian surface epithelial cells, such as immortalized IOSE models (e.g., IOSE80), would also provide important information regarding selective cytotoxicity, therapeutic index, and potential off-target toxicity. An additional limitation of the present study is the lack of direct experimental validation of the molecular targets predicted through in silico bioinformatics analyses, which should be addressed in future mechanistic and protein-level investigations. Accordingly, the target prediction, PPI network construction, and GO/KEGG enrichment results should be interpreted as hypothesis-generating rather than as definitive mechanistic evidence. Beyond these methodological limitations, important translational considerations should be noted. Despite its well-documented anticancer activity in vitro, curcumin is characterized by poor aqueous solubility and low systemic bioavailability, which limit the direct clinical translatability of the concentrations used in the present study. Accordingly, the observed synergistic effects should be interpreted as experimental exposure-dependent phenomena rather than as clinically achievable dosing conditions. Future studies employing optimized formulations, delivery systems, or curcumin analogs with improved pharmacokinetic properties will be required to enhance in vivo relevance. Moreover, although ROS modulation experiments indicate that oxidative stress contributes to combination-induced cytotoxicity, the attenuation of etoposide-induced ROS levels by curcumin suggests redox modulation rather than simple ROS amplification, warranting cautious interpretation of redox-dependent mechanisms. Taken together, these considerations underscore that the present findings are hypothesis-generating and support further preclinical investigation rather than immediate clinical application.

## 5. Conclusions

This study provides integrated quantitative evidence supporting a synergistic interaction between etoposide and curcumin in ovarian cancer models. Using complementary analytical approaches, the combination demonstrated enhanced biological activity compared with single-agent treatments, including increased apoptotic cell death, coordinated modulation of apoptosis-related gene expression, and suppression of inflammatory and angiogenic mediators. The magnitude and consistency of these effects were cell line-dependent, with more robust synergy observed in OVCAR3 cells and a more moderate, dose-restricted response in SKOV3 cells.

The enhanced biological effects observed in two-dimensional cultures were preserved in a 3D tumor spheroid model, where combination treatment resulted in pronounced spheroid shrinkage, reduced viability, and disruption of spheroid architecture. ROS modulation experiments indicated that oxidative stress contributes to combination-induced cytotoxicity; however, curcumin attenuated etoposide-induced ROS levels, suggesting redox modulation rather than ROS amplification. Partial reversal of cytotoxicity by NAC further supports the involvement of both ROS-dependent and ROS-independent mechanisms.

In silico target prediction and pathway enrichment analyses identified apoptosis- and p53-associated signaling networks as potential components of the observed response. These findings should be interpreted as hypothesis-generating, particularly in SKOV3 cells where p53 signaling is known to be impaired. Overall, the present results support further preclinical evaluation of the etoposide–curcumin combination using advanced in vivo models to better define its translational potential in ovarian cancer.

## Data Availability

The raw data supporting the conclusions of this article will be made available by the authors on request.

## Supplementary Materials

The following supporting information can be downloaded at: https://www.mdpi.com/article/10.3390/biomedicines14030509/s1. Figure S1. Dose-dependent effects of etoposide on ovarian cancer cell viability.

## Abbreviations

3D	Three-dimensional
ANOVA	Analysis of variance
ATP	Adenosine triphosphate
BAX	BCL2-associated X protein
BCL2	B-cell lymphoma 2
CASP3	Caspase-3
CI	Combination Index
DCFH-DA	2′,7′-Dichlorodihydrofluorescein diacetate
DMSO	Dimethyl sulfoxide
ELISA	Enzyme-linked immunosorbent assay
ESR1	Estrogen receptor 1
Fa	Fraction affected
FDR	False discovery rate
FITC	Fluorescein isothiocyanate
GAPDH	Glyceraldehyde-3-phosphate dehydrogenase
GO	Gene Ontology
HIF-1	Hypoxia-inducible factor 1
IC_50_	Half-maximal inhibitory concentration
IL-6	Interleukin-6
KEGG	Kyoto Encyclopedia of Genes and Genomes
MAPK1	Mitogen-activated protein kinase 1
MFI	Mean fluorescence intensity
MTT	3-(4,5-dimethylthiazol-2-yl)-2,5-diphenyltetrazolium bromide
NAC	*N*-acetyl-*L*-cysteine
OVCAR3	Human ovarian adenocarcinoma cell line
PBS	Phosphate-buffered saline
PI	Propidium iodide
PI3K–Akt	Phosphoinositide 3-kinase–Akt signaling pathway
PPI	Protein–protein interaction
qRT-PCR	Quantitative real-time polymerase chain reaction
ROS	Reactive oxygen species
SEM	Standard error of the mean
SKOV3	Human ovarian adenocarcinoma cell line
STAT3	Signal transducer and activator of transcription 3
TNF-α	Tumor necrosis factor alpha
TP53	Tumor protein p53
ULA	Ultra-low attachment
VEGF	Vascular endothelial growth factor
VEGFA	Vascular Endothelial Growth Factor A
