# Supplementary material for: Synergistic Antitumor Effects of Etoposide and Curcumin in Ovarian Cancer Cells
Source: Biomedicines. 2026 Feb 25;14(3):509. doi: 10.3390/biomedicines14030509 (PMC13023443; doi:10.3390/biomedicines14030509)
Supplement: Supplementary file 1 [file biomedicines-14-00509-s001.zip › biomedicines-4143768-supplementary.pdf]

Figure S1. Dose-dependent effects of etoposide on ovarian cancer cell viability.

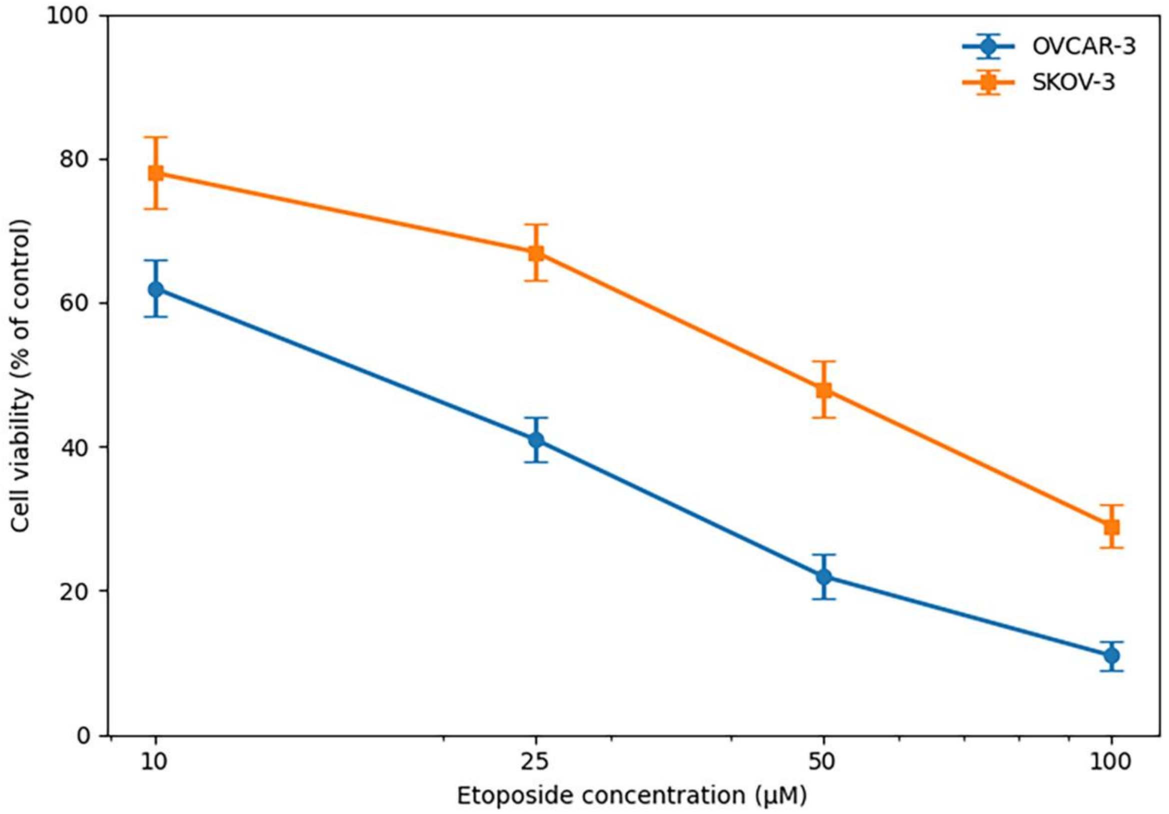

OVCAR-3 and SKOV-3 cells were treated with increasing concentrations of etoposide (10–100 μM), and cell viability was assessed relative to untreated control cells. Data are expressed as percentage of control viability and presented as mean ± SEM from at least three independent experiments. OVCAR-3 cells exhibited greater sensitivity to etoposide compared with SKOV-3 cells across all tested concentrations.
